# Supplementary figures and images for: Transcription-induced formation of extrachromosomal DNA during yeast ageing
Source: PLoS Biol. 2019 Dec 3;17(12):e3000471. doi: 10.1371/journal.pbio.3000471 (PMC6890164; doi:10.1371/journal.pbio.3000471)

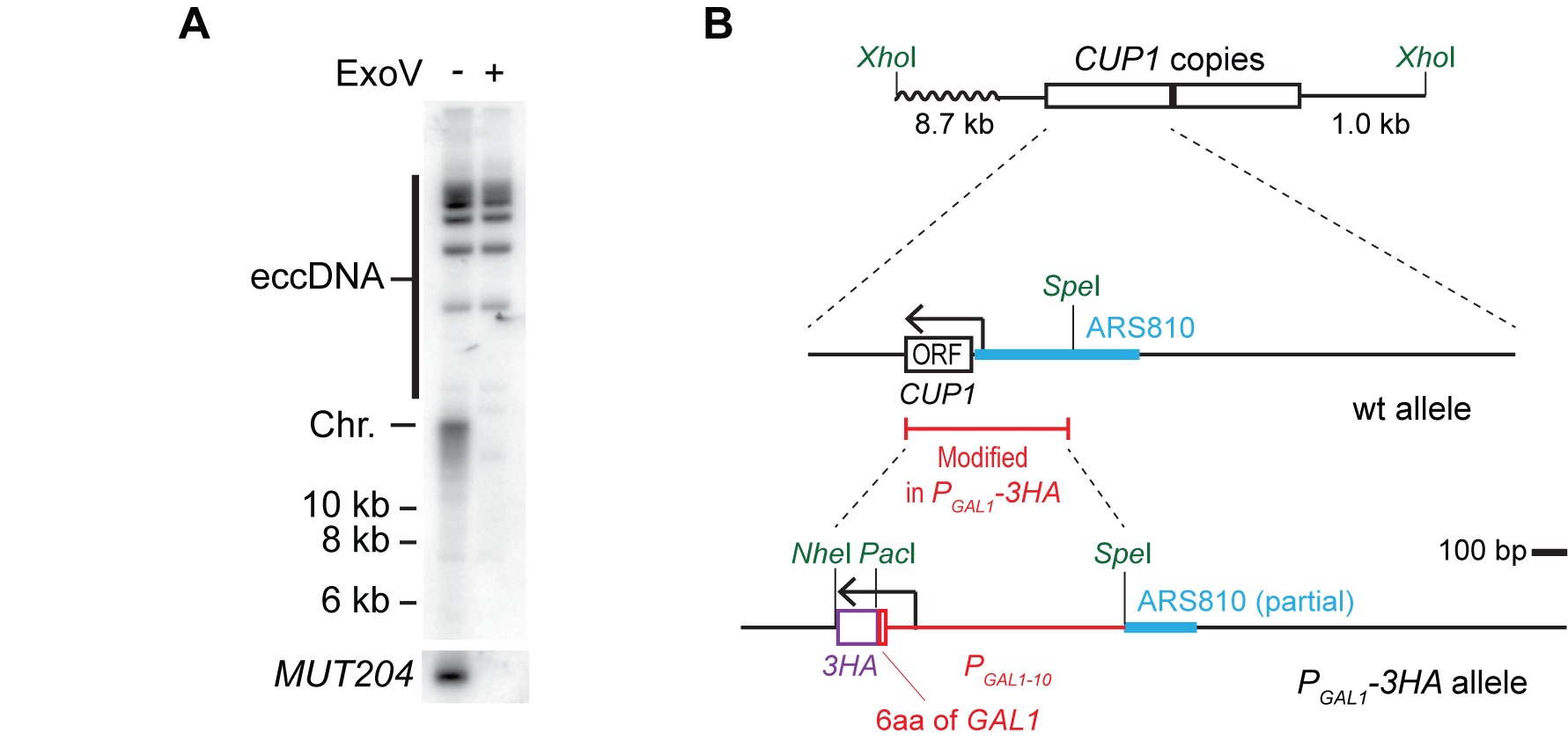

Supplement: S1 Fig — (A) Cells were aged in the presence of CuSO4, and genomic DNA purified before being split in 2 aliquots. Both aliquots were digested with XhoI, and one was additionally digested with ExoV (aka RecBCD) in the presence of 1 mM ATP. After purification, DNA was separated on a 1% agarose gel and probed for the CUP1 locus, then stripped and reprobed for MUT204, a single copy intergenic region on Chromosome III. (B) Schematic of the CUP1 locus: detailed view of a single CUP1 repeat, and map of differences between the wild-type and PGAL1-3HA allele. In this allele, the CUP1 ORF and CUP1 promoter along with part of the annotated ARS810 sequence are removed and replaced by a fused ORF consisting of 3HA and 6 amino acids of GAL1 and the PGAL1-10 promoter. Restriction sites are shown for orientation, the NheI site was added during construction between the CUP1 stop codon and start of the 3’ UTR. The data underlying this figure may be found in S1 Raw Images. ExoV, exonuclease V; ORF, open reading frame (TIF) [file pbio.3000471.s001.tif]

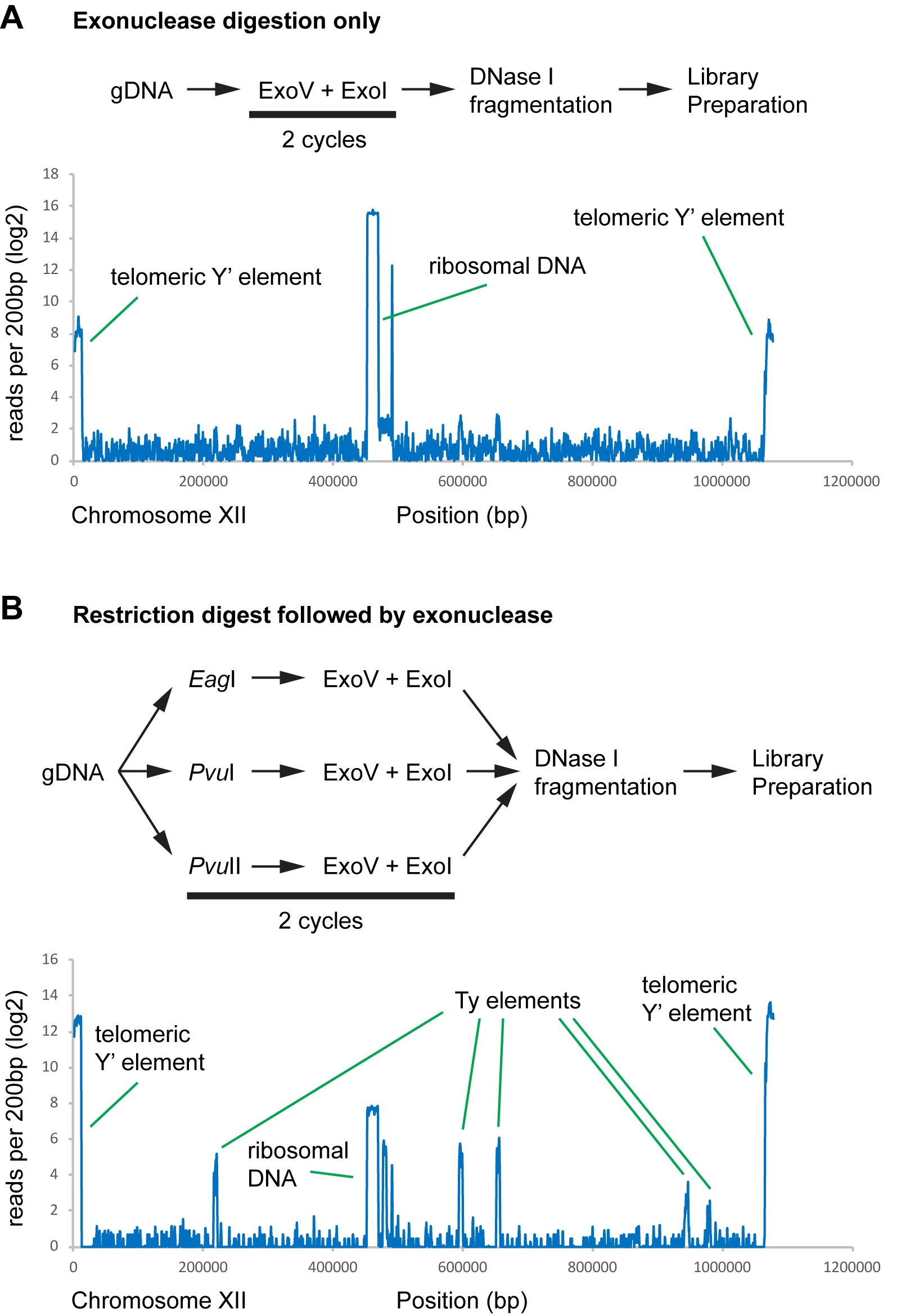

Supplement: S2 Fig — Plots of sequencing reads summed in 200 bp bins across Chromosome XII. (A) Genomic DNA from cells aged for 48 hours in SD media was digested directly with ExoV + ExoI only prior to library preparation. ExoI is included in these reactions to remove short single-stranded products of ExoV digestion. (B) Genomic DNA from cells aged for 48 hours in SD media was split, digested with 3 different enzymes followed by ExoV + ExoI before library preparation. Note the prominent Ty-element peaks in panel B that are not detectable in panel A. The data underlying this figure may be found in S1 Data. eccDNA, extrachromosomal circular DNA; ExoV, exonuclease V; REC-seq, restriction-digested extrachromosomal circular DNA sequencing (TIF) [file pbio.3000471.s002.tif]

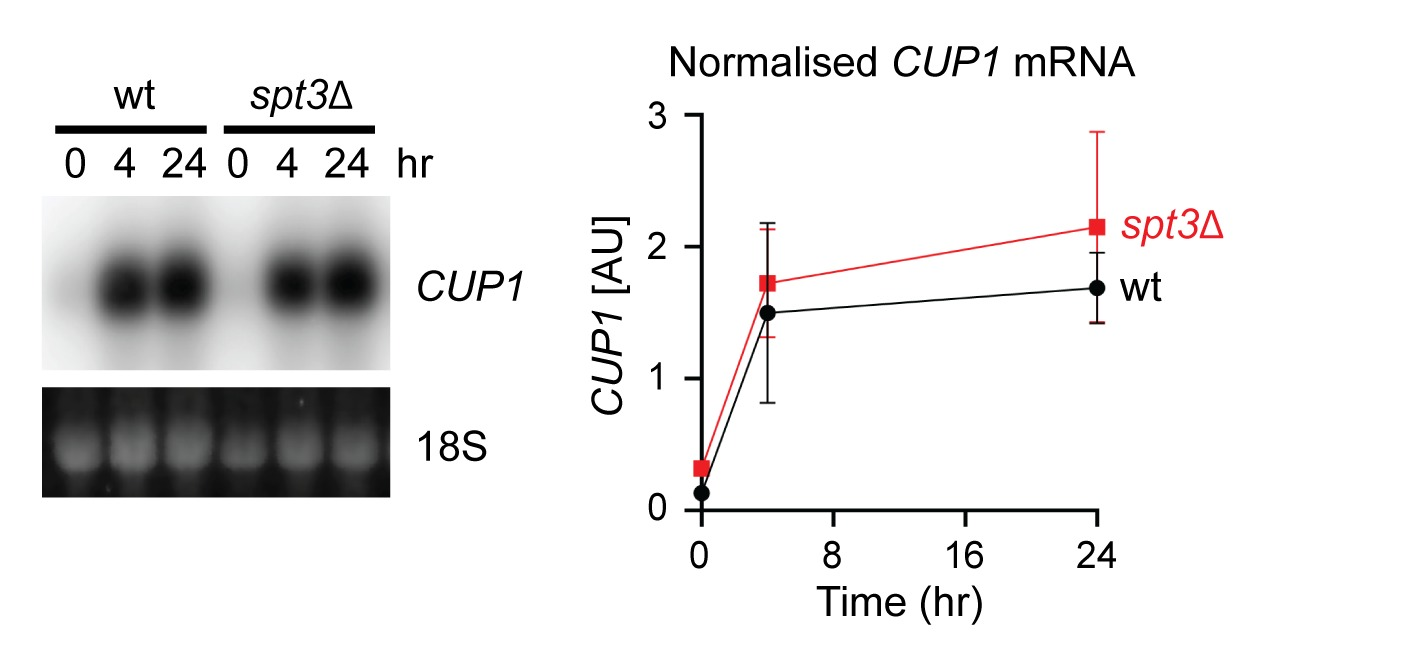

Supplement: S3 Fig — MEP wild-type and spt3Δ cells growing in SD media were induced with 1 mM CuSO4 and cells harvested after 0, 4, and 24 hours. Total RNA was separated on a glyoxal gel and probed for CUP1 ORF, ethidium stained 18S ribosomal RNA is shown as a loading control. Error bars show standard deviation, n = 3. The data underlying this figure may be found in S1 Data and S1 Raw Images. MEP, mother enrichment program (TIF) [file pbio.3000471.s003.tif]

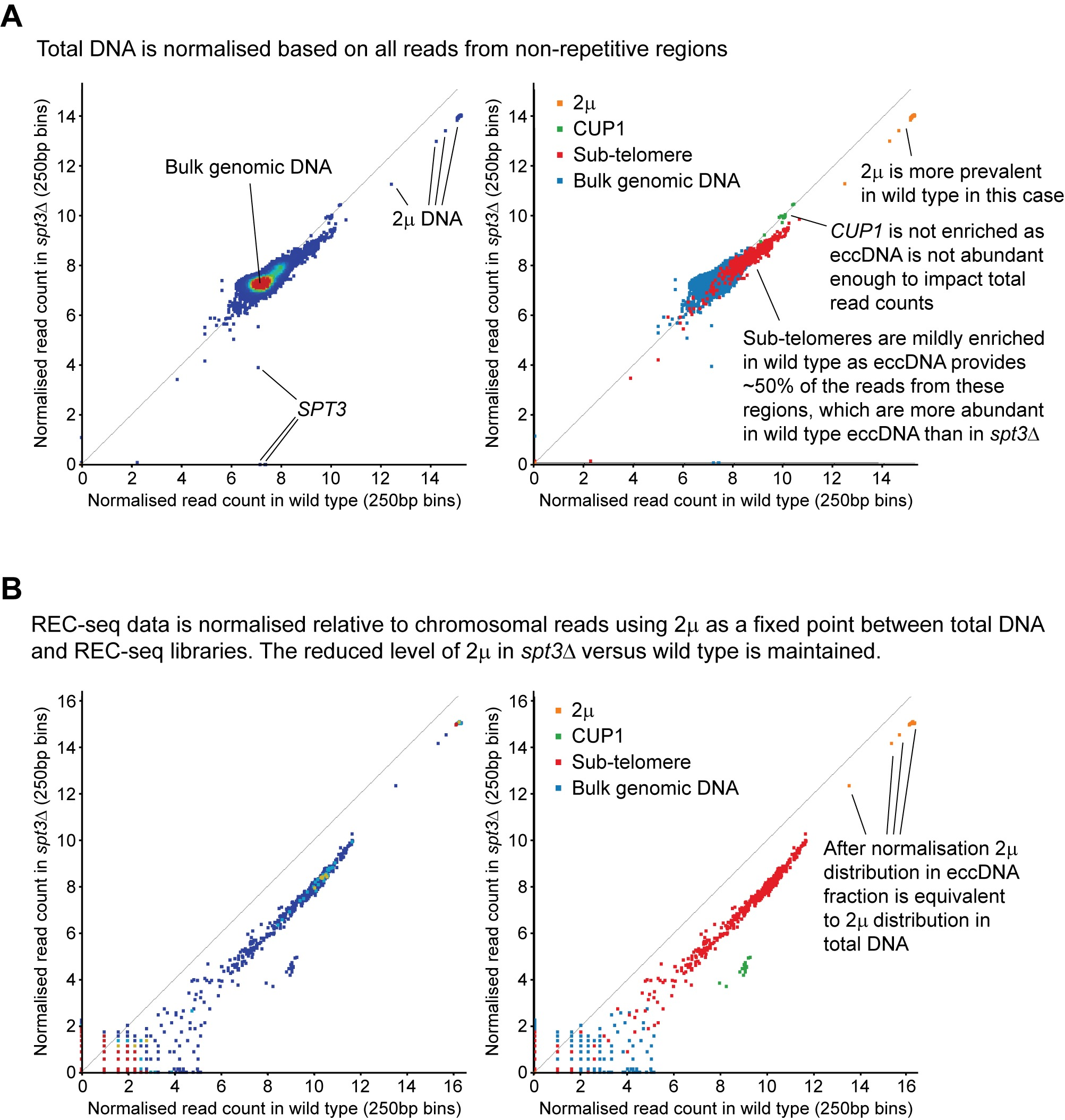

Supplement: S4 Fig — (A) Comparison of total DNA sequencing in aged wild-type and spt3Δ cells split in 250 bp bins across the genome. Two graphs of the same data are shown, one as a density plot (left), the other highlighting prominent features (right). Most bins representing genomic DNA are on the centre line, significant outliers are labelled—SPT3 locus is absent in spt3Δ cells, 2μ is highly abundant but in this case slightly more prevalent in the wild-type sample. The subtelomeric regions are shifted towards wild type as the high levels of eccDNA produced from these loci adds to the read count even in total DNA. (B) Comparison of eccDNA sequencing from the same 2 samples as in panel A. The normalisation ensures that the ratio of 2μ reads between wild-type and spt3Δ is maintained, even though all circles are under-represented in the spt3Δ sample, a difference that would not be detected without such normalisation. The data underlying this figure may be found in S1 Data. eccDNA, extrachromosomal circular DNA (TIF) [file pbio.3000471.s004.tif]

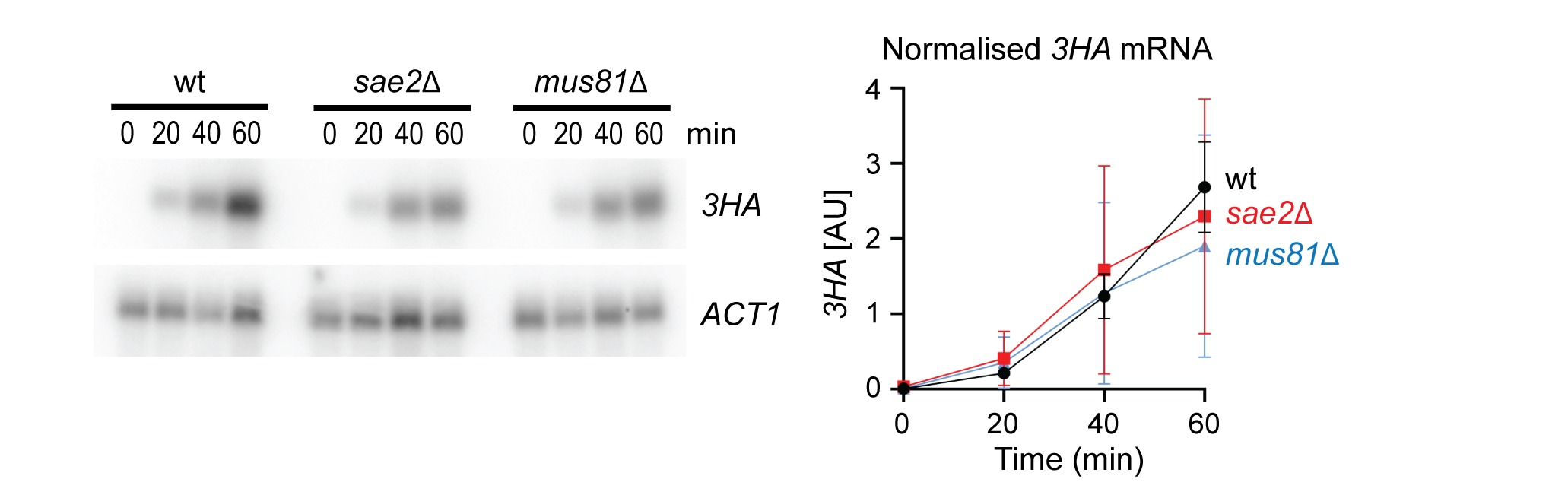

Supplement: S5 Fig — PGAL1-3HA cells in BY4741 background with no deletion, sae2Δ or mus81Δ growing in YP raffinose media were induced with 2% galactose and cells harvested after 0, 20, 40, and 60 minutes. Total RNA was separated on a glyoxal gel and probed for CUP1 ORF, followed by the ACT1 ORF as a loading control. Error bars show standard deviation, n = 2. The data underlying this figure may be found in S1 Data and S1 Raw Images. (TIF) [file pbio.3000471.s005.tif]
