# Supplementary material for: Transcription-induced formation of extrachromosomal DNA during yeast ageing
Source: PLoS Biol. 2019 Dec 3;17(12):e3000471. doi: 10.1371/journal.pbio.3000471 (PMC6890164; doi:10.1371/journal.pbio.3000471)
Supplement: S1 Raw Images — (PDF) [file pbio.3000471.s011.pdf]

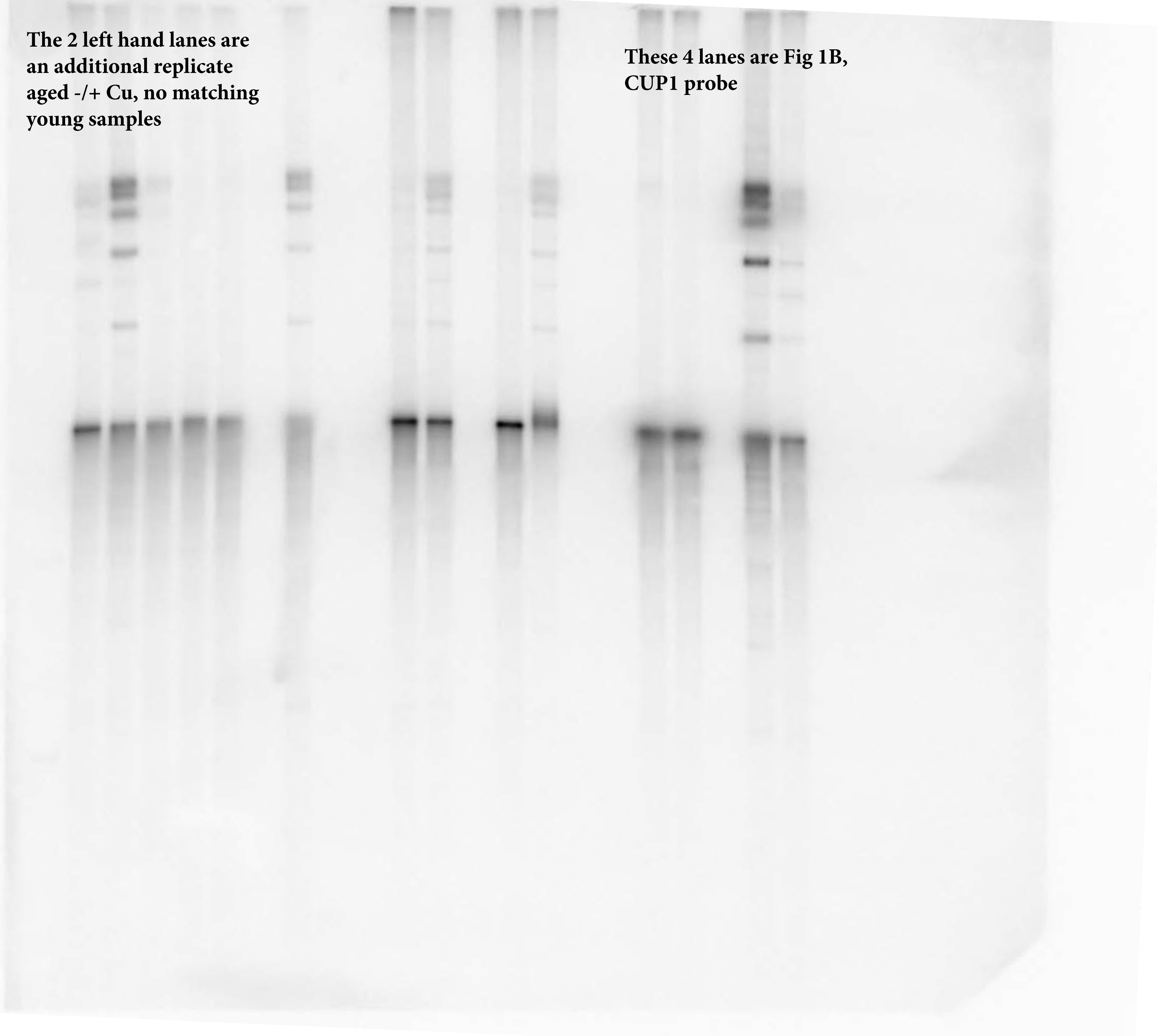
The 2 left hand lanes are an additional replicate aged -/+ Cu, no matching young samples
These 4 lanes are Fig 1B, CUP1 probe

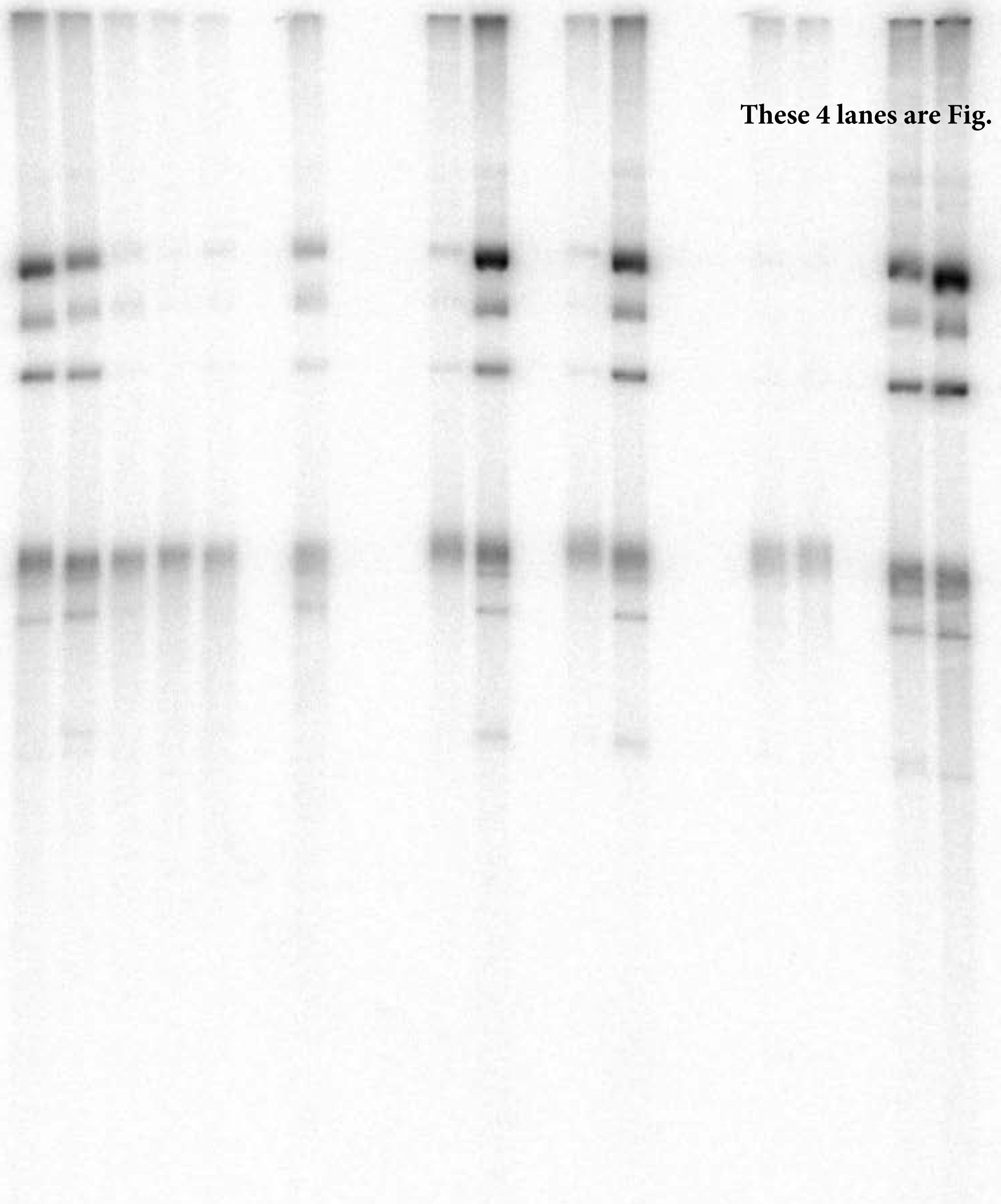

These 4 lanes are Fig. 1B, rDNA probe

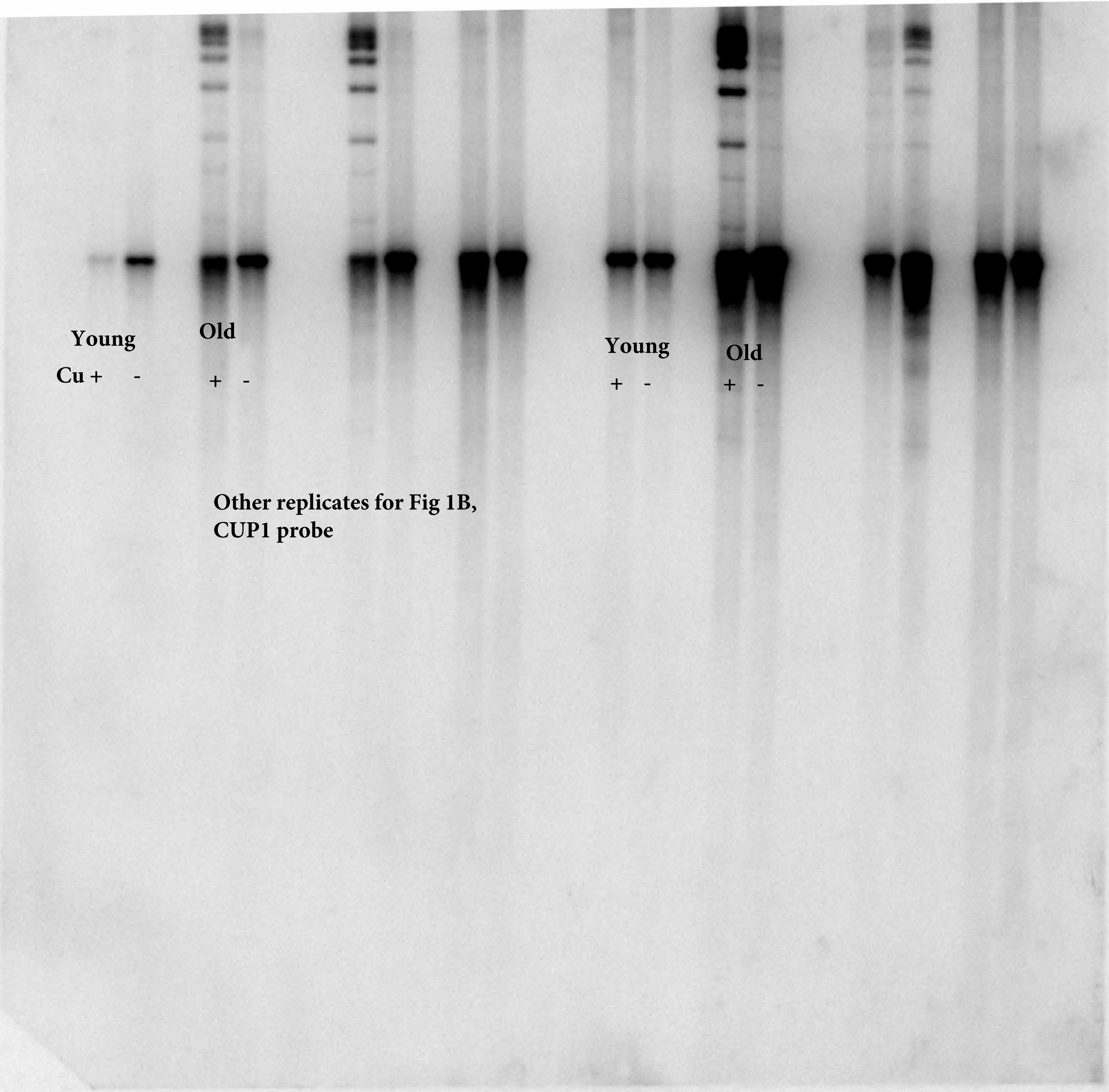
Young
Old
Cu + -
+ -
Young
Old
+ -
+ -
Other replicates for Fig 1B,
CUP1 probe

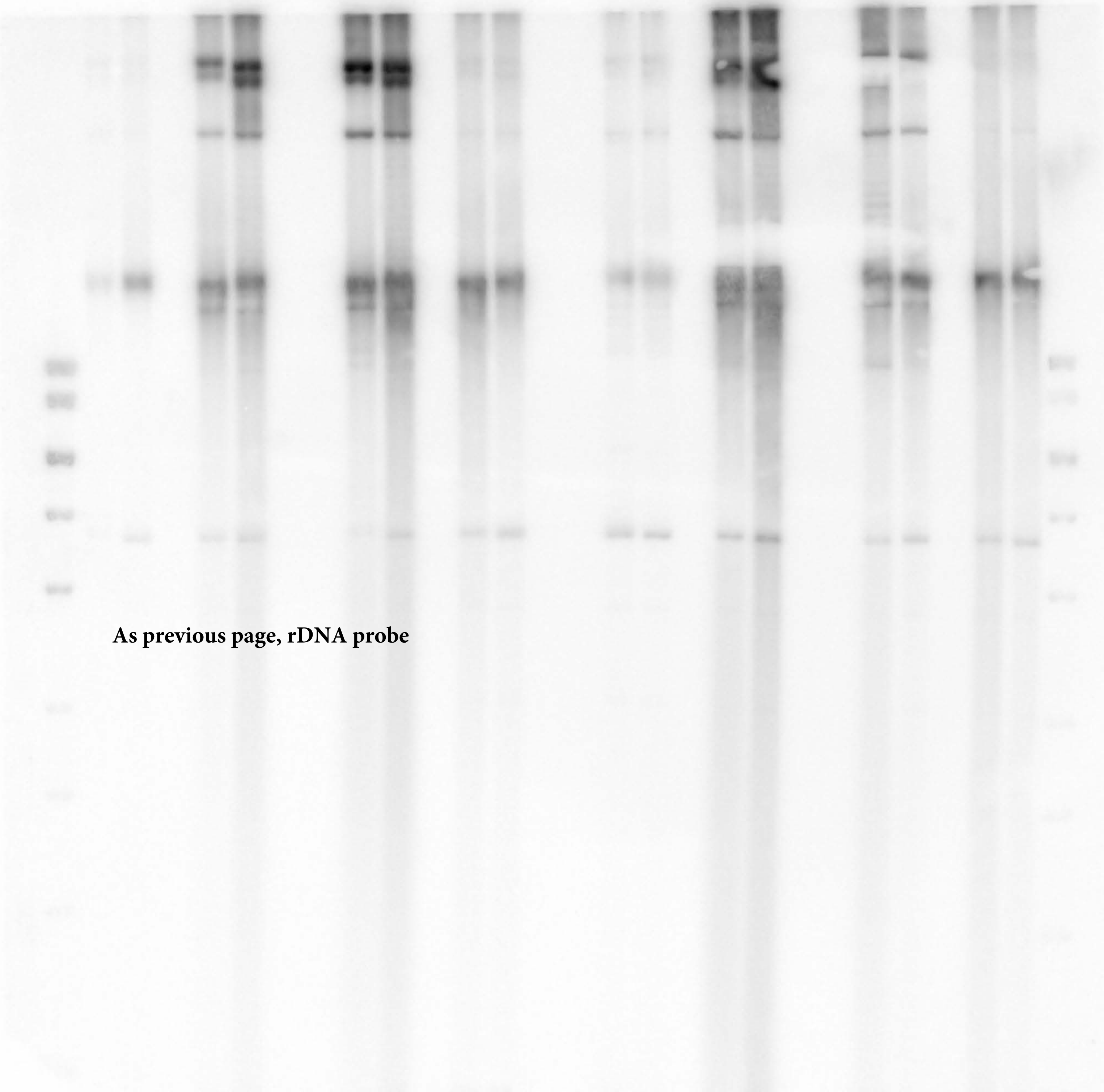

As previous page, rDNA probe

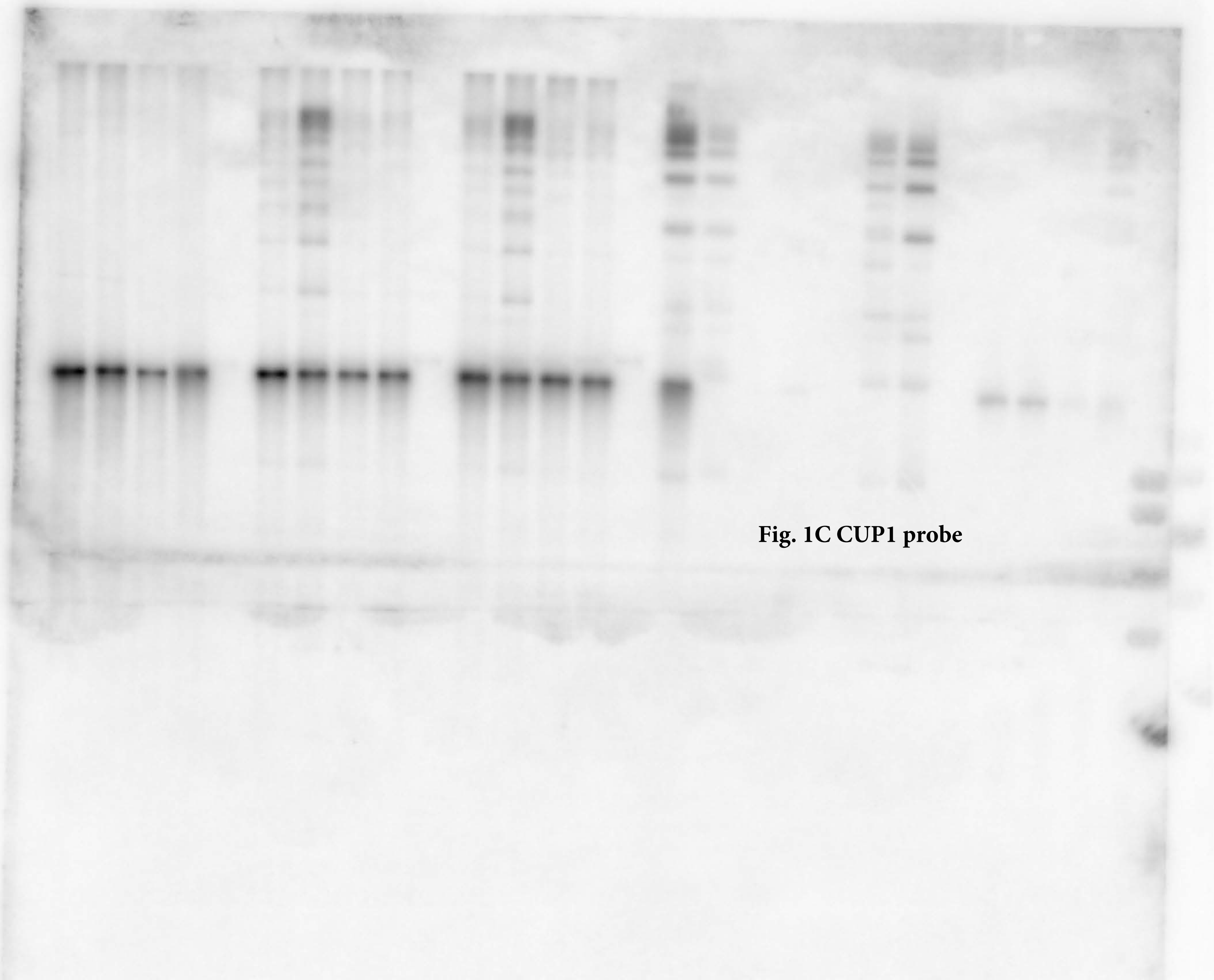

Fig. 1C CUP1 probe

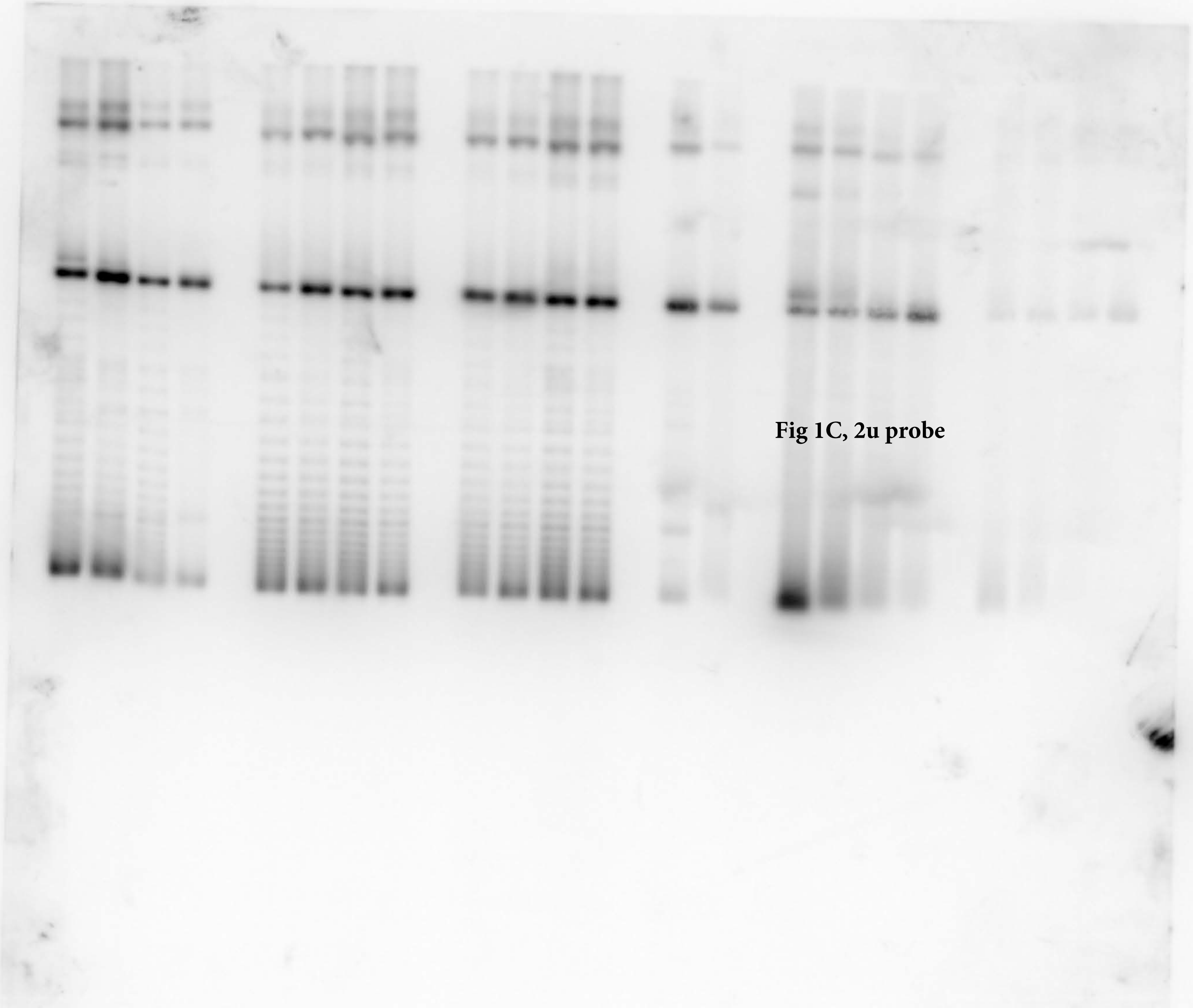
Fig 1C, 2u probe

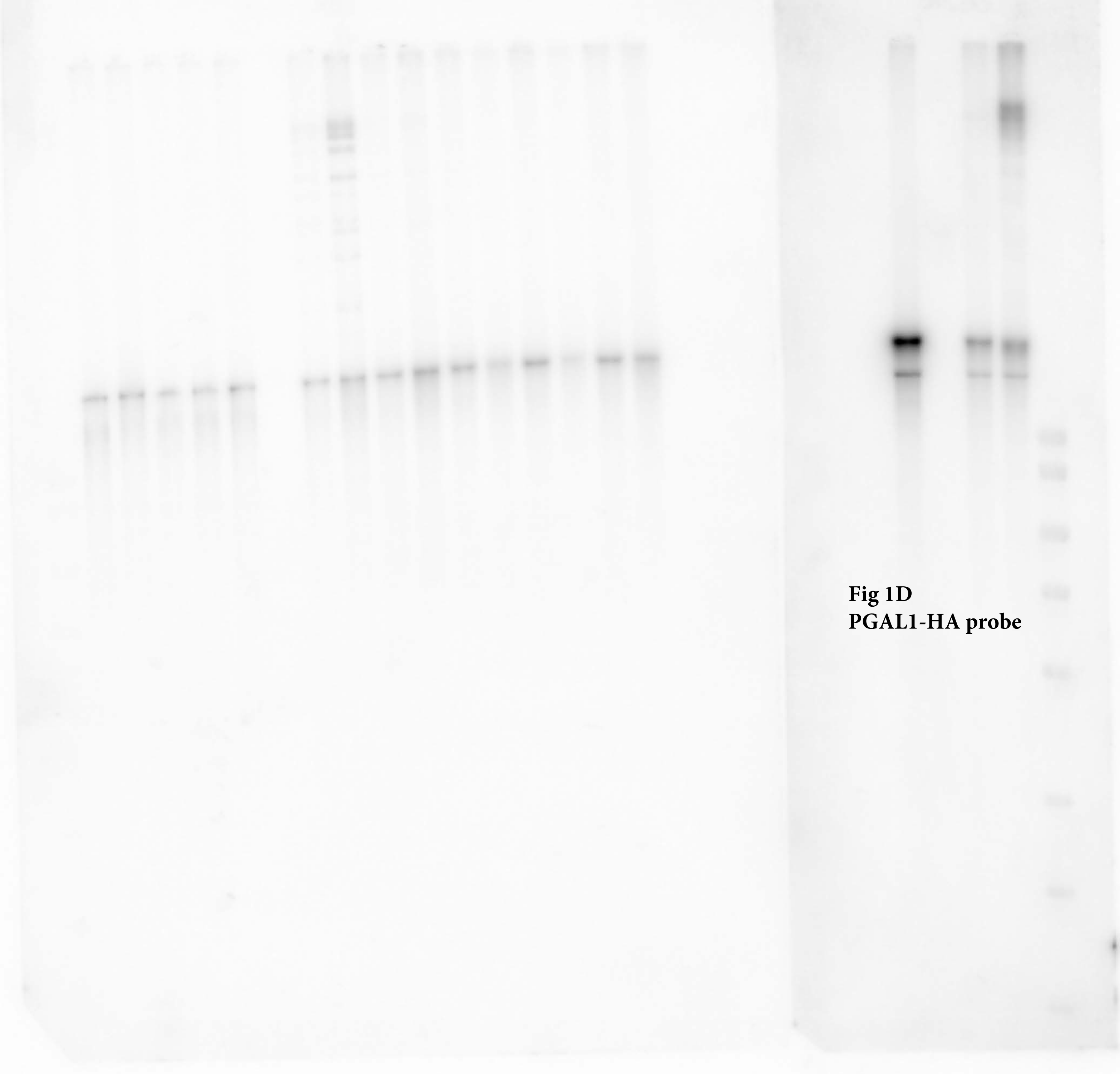

Fig 1D
PGAL1-HA probe

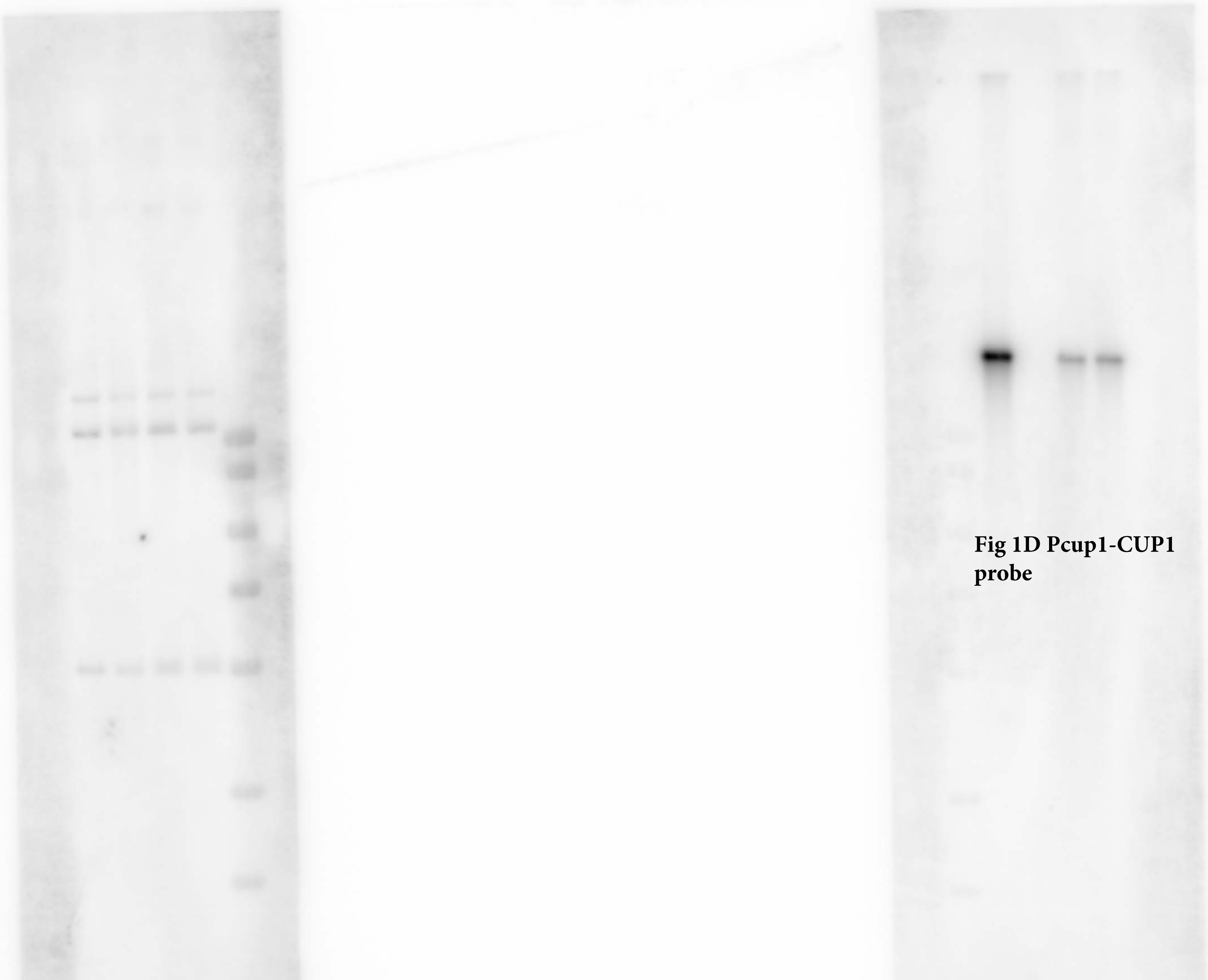

Fig 1D Pcup1-CUP1
probe

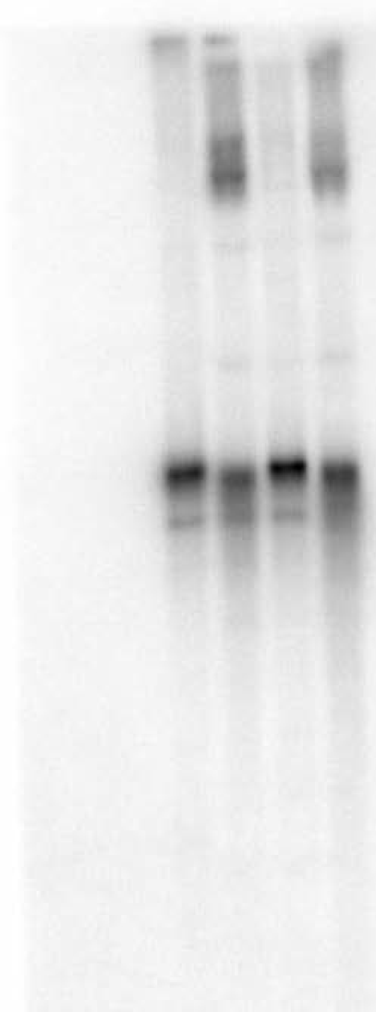

Fig 1D other 2 replicates of aged samples
lanes are glu, gal, glu, gal
Probed with Pgal1-3HA

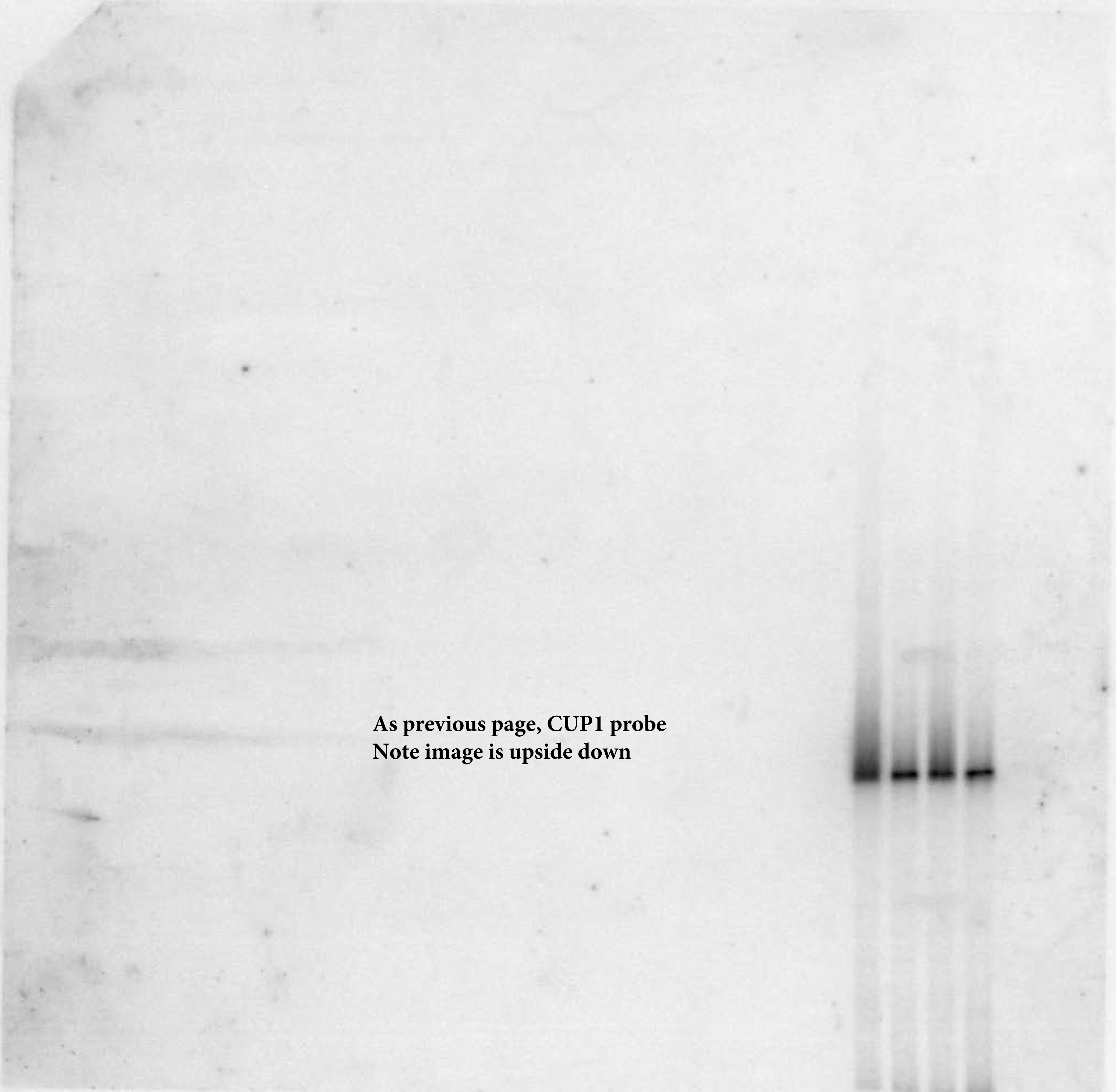

As previous page, CUP1 probe
Note image is upside down

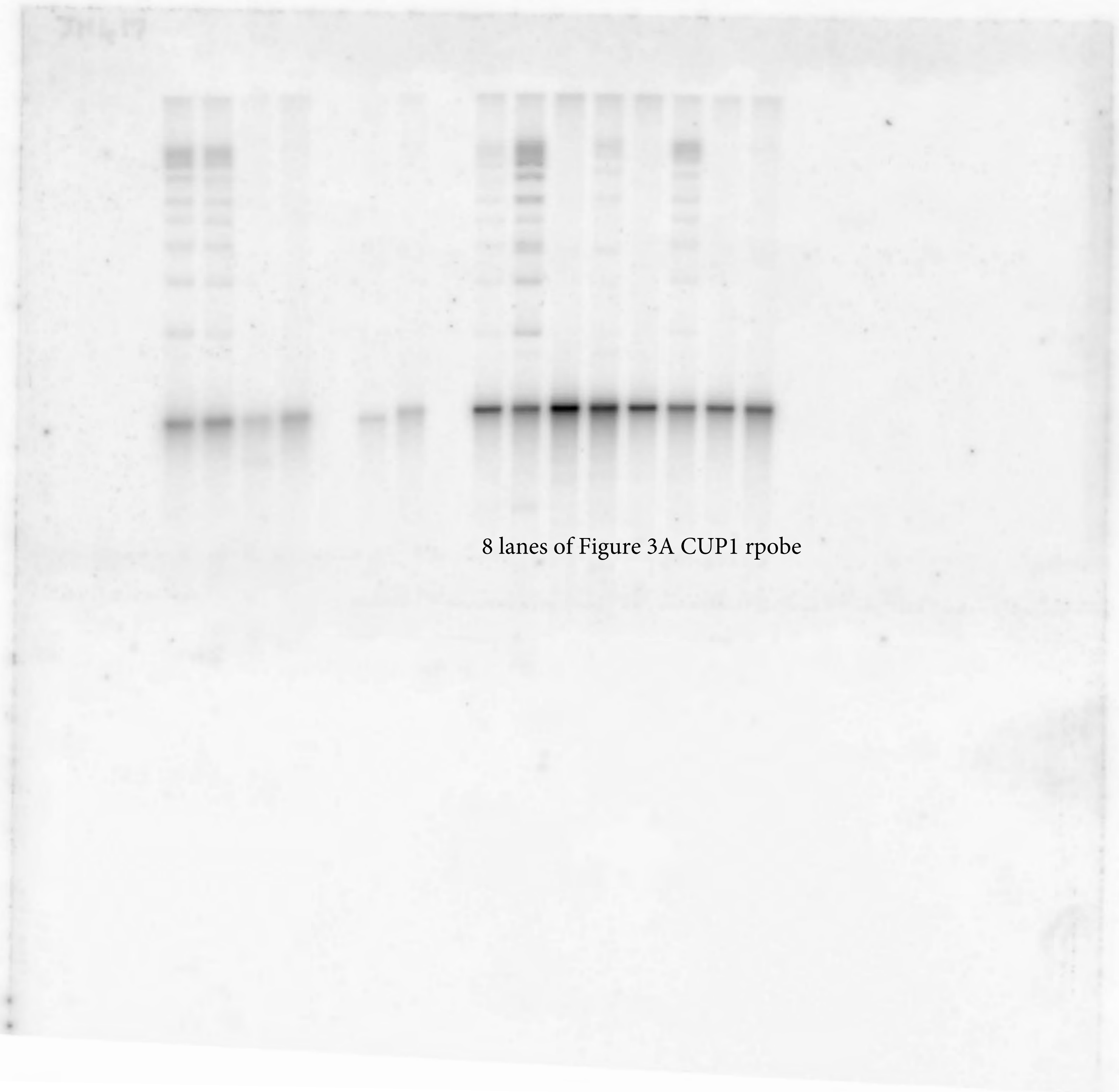
8 lanes of Figure 3A CUP1 rpobe

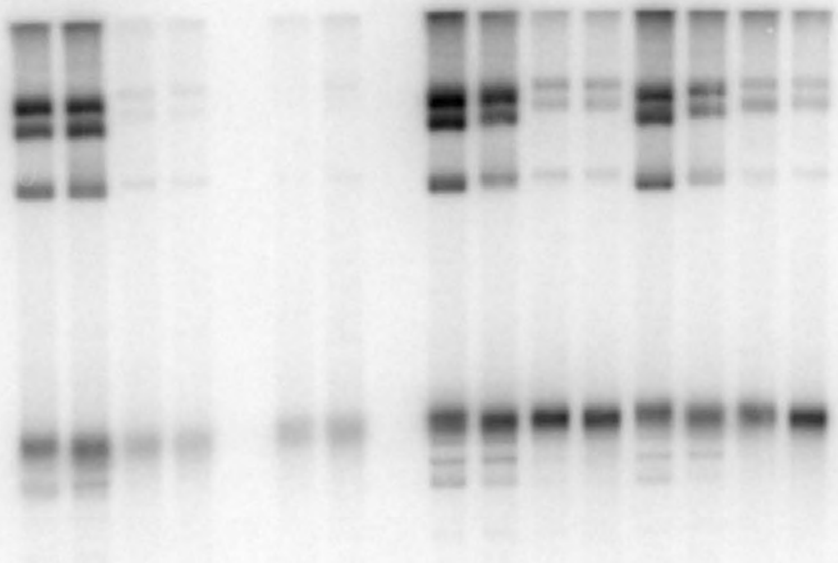

As previous, rDNA probe

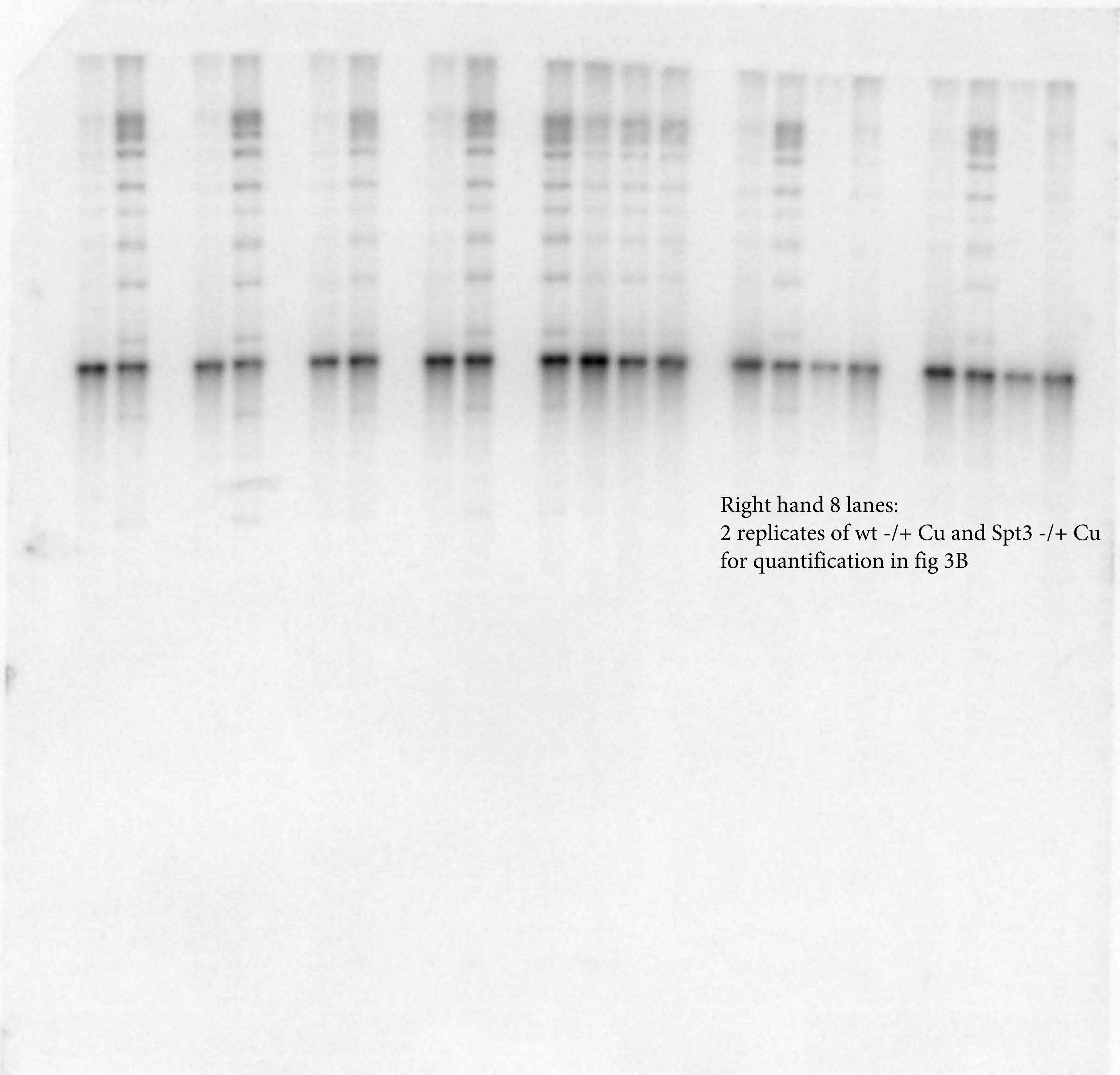
Right hand 8 lanes:
2 replicates of wt -/+ Cu and Spt3 -/+ Cu
for quantification in fig 3B

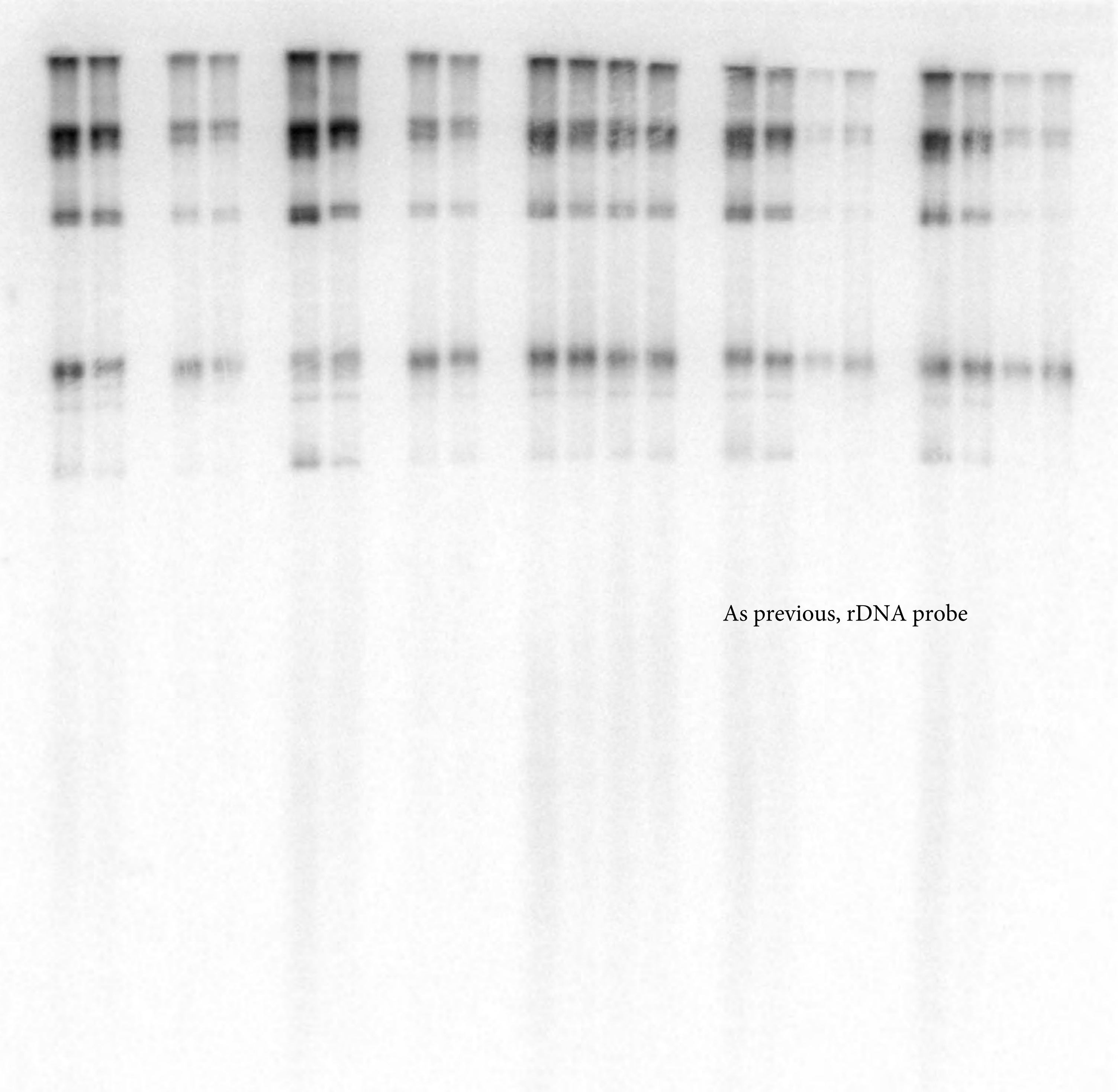

As previous, rDNA probe

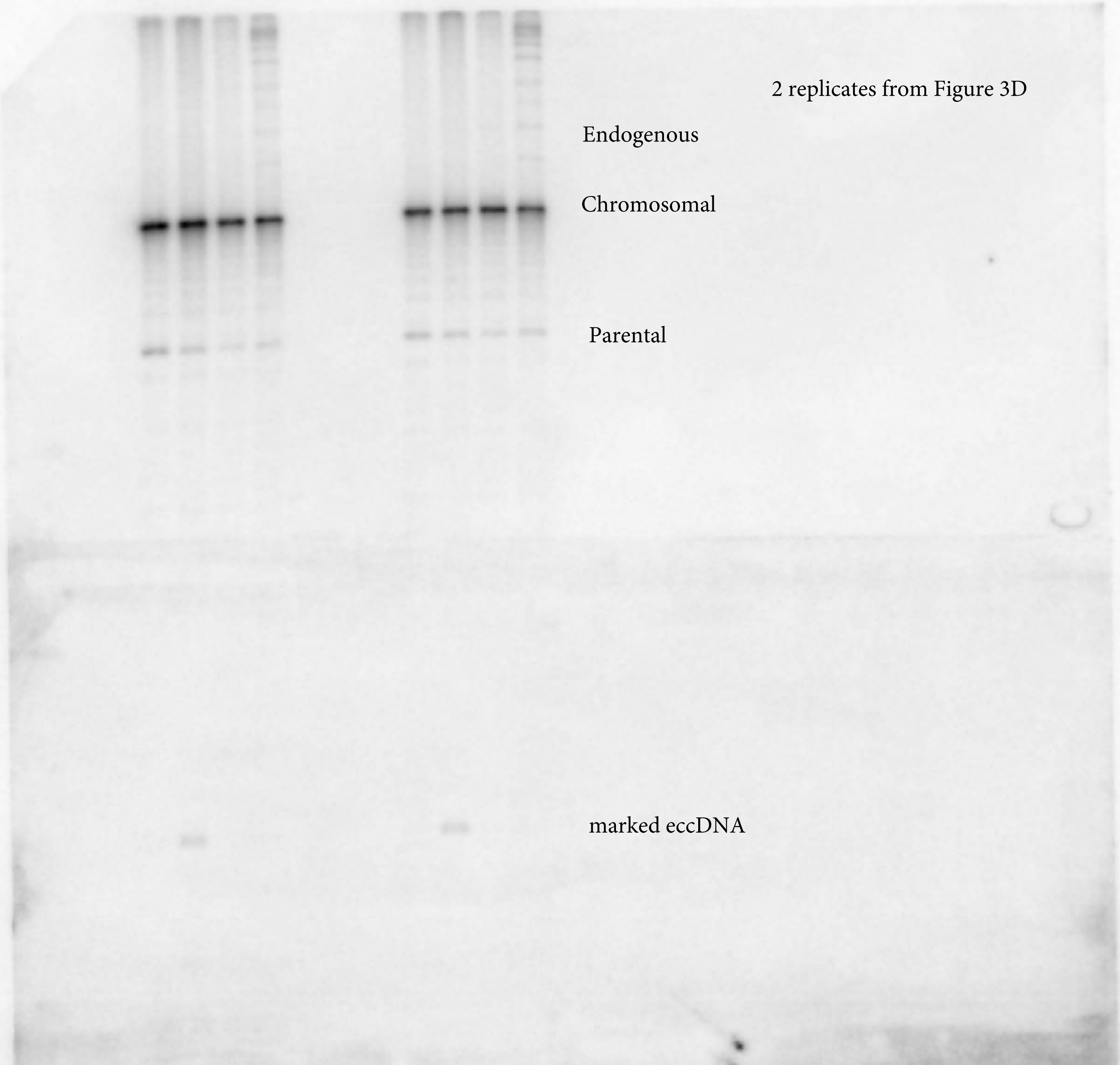

2 replicates from Figure 3D
Endogenous
Chromosomal
Parental
marked eccDNA

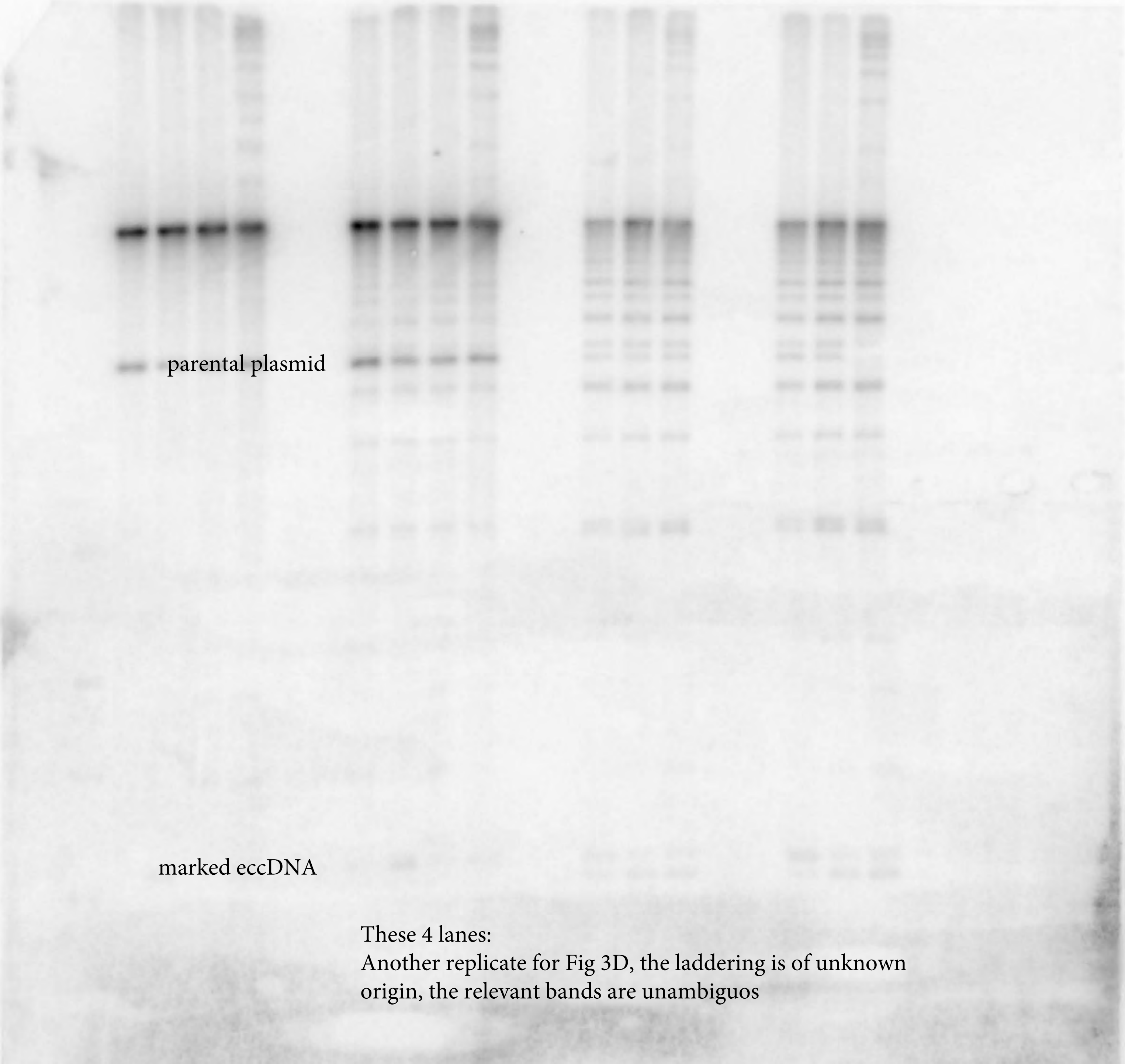
parental plasmid
marked eccDNA
These 4 lanes:
Another replicate for Fig 3D, the laddering is of unknown origin, the relevant bands are unambiguos

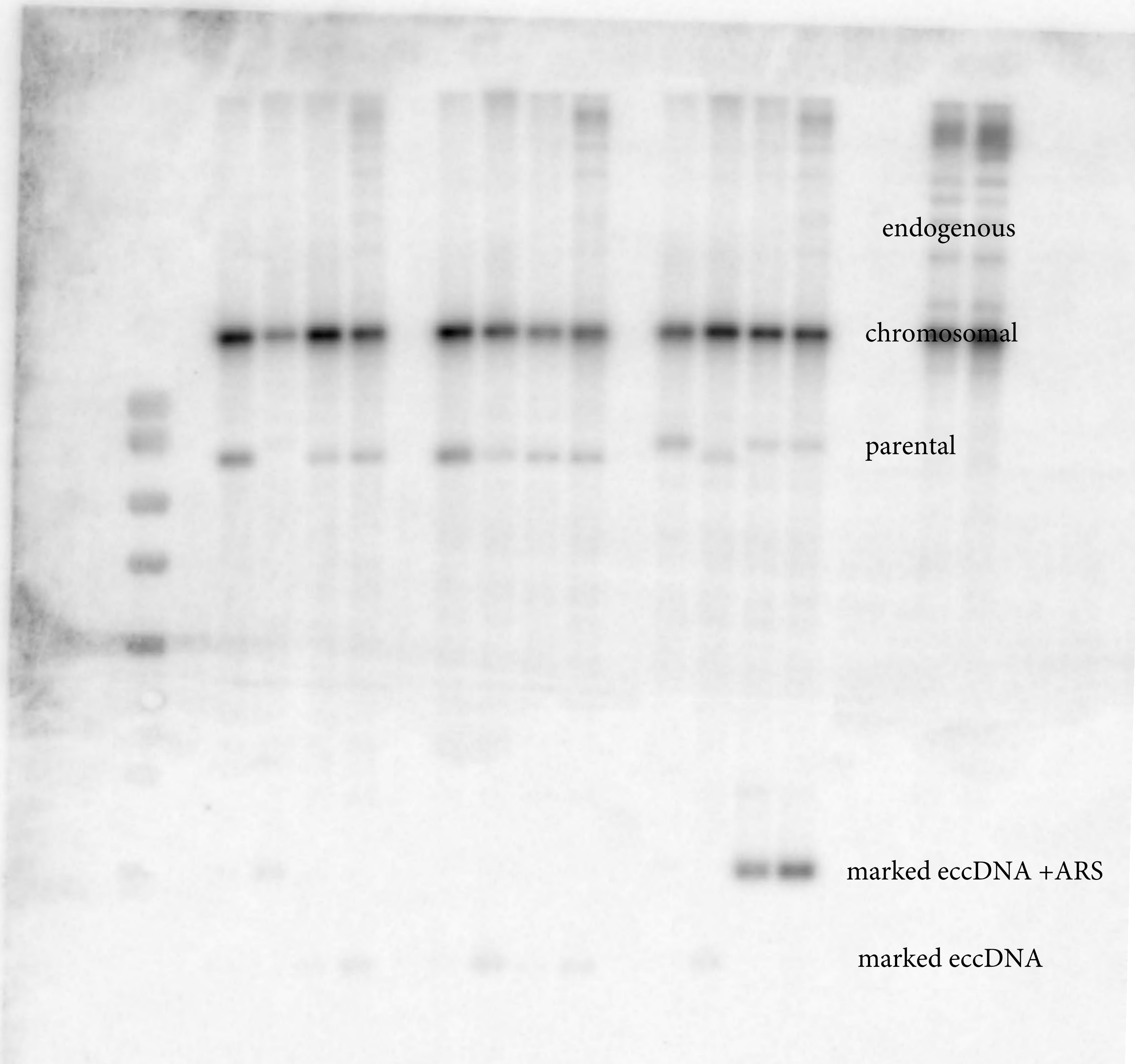

Note - lanes 2 and 10 have been swapped (lane counts do not include ladders and blanks)

Lanes 1-4 replicate for Fig 3D
Lanes 9-12 replicate for Fig 3E

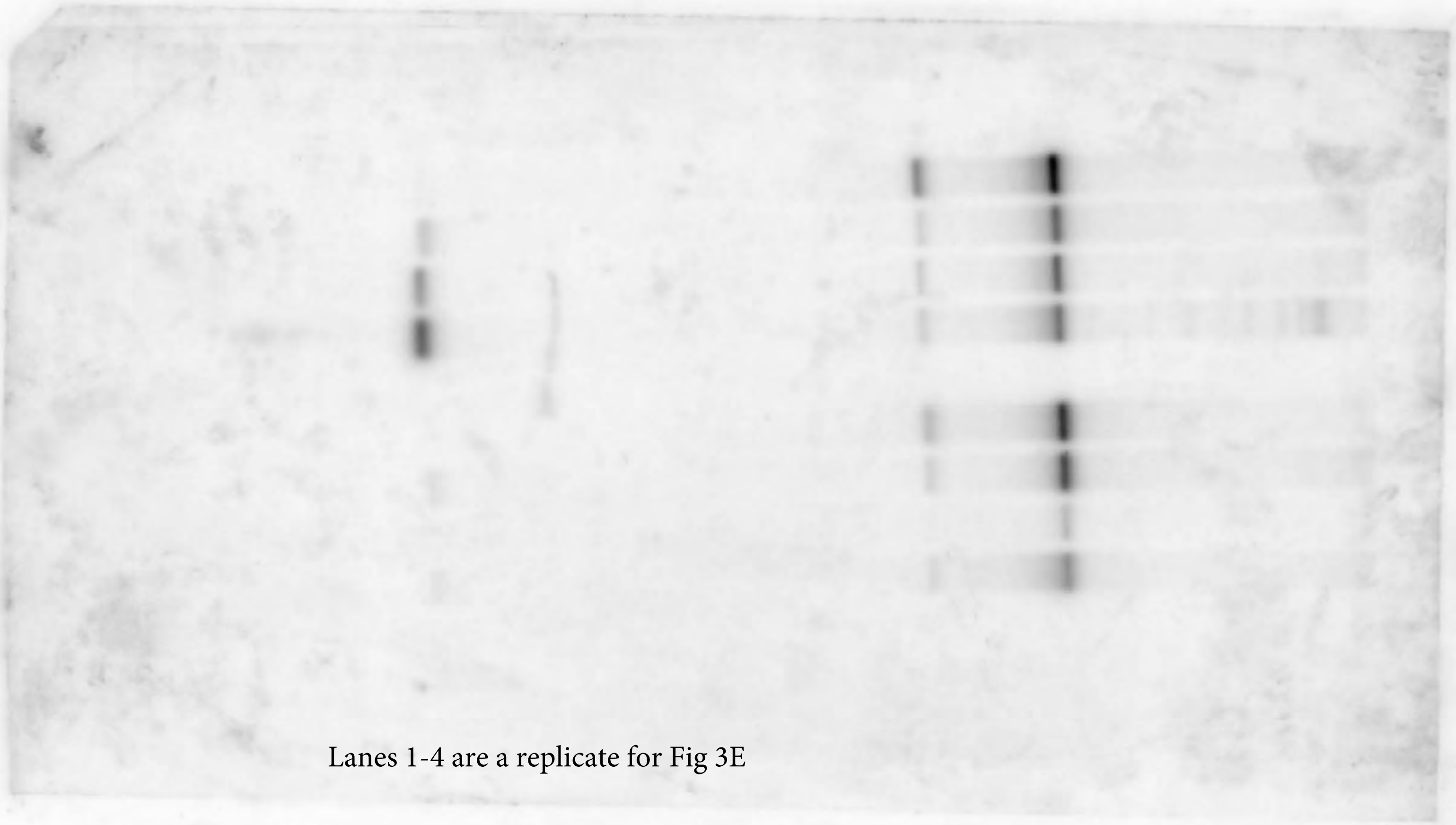
Lanes 1-4 are a replicate for Fig 3E

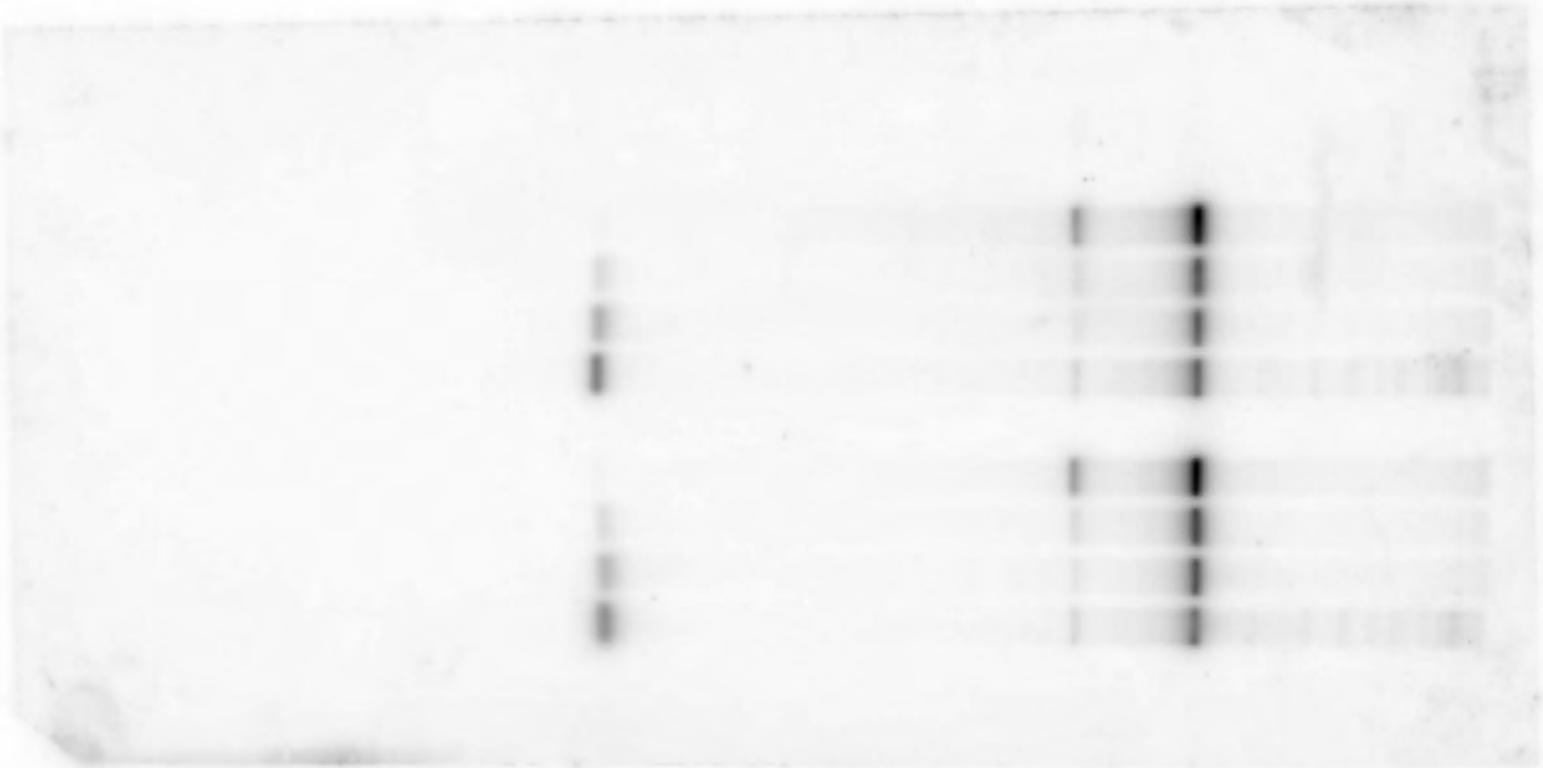

2 replicates for Fig 3E

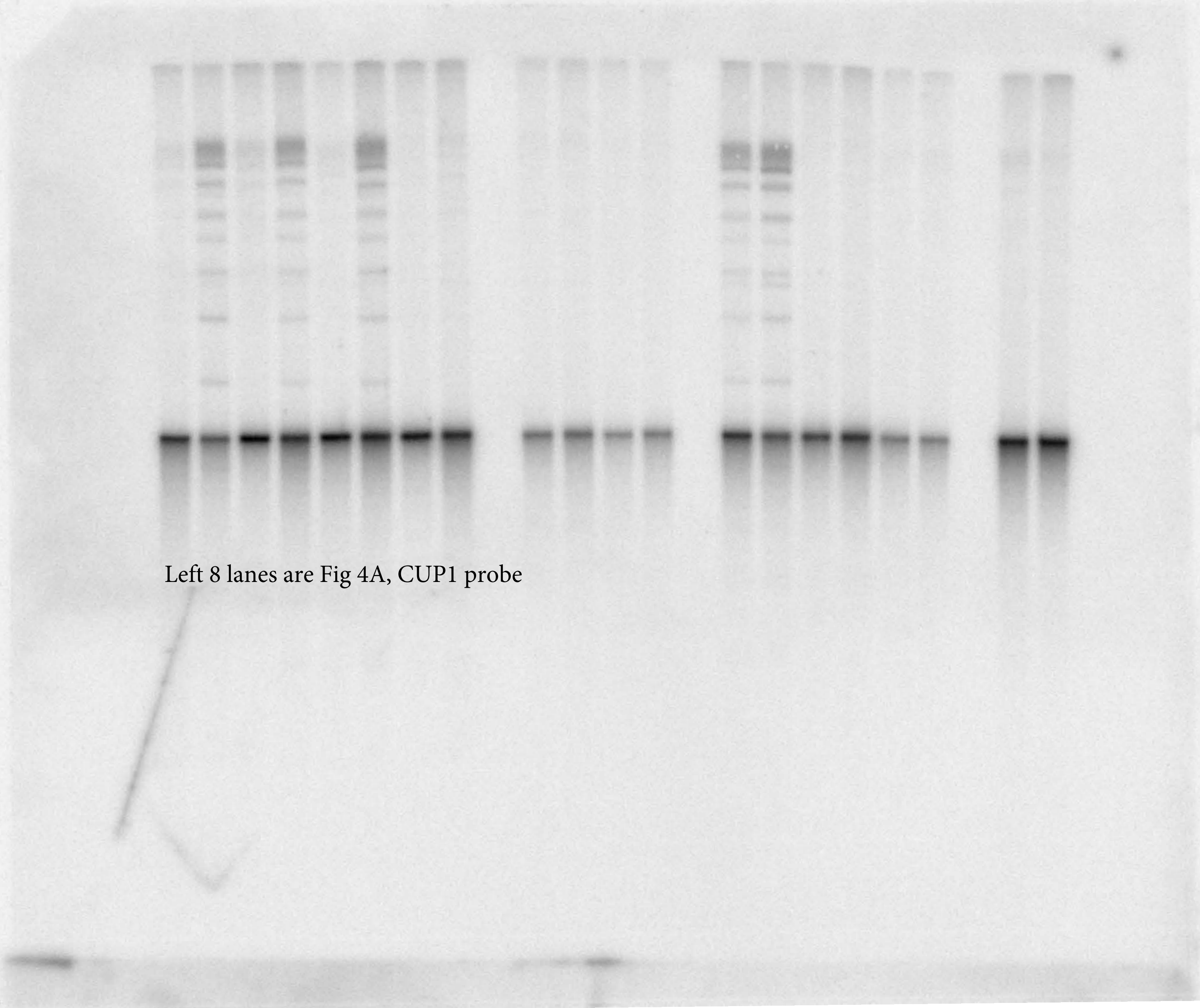

Left 8 lanes are Fig 4A, CUP1 probe

As above, rDNA probe, upside down

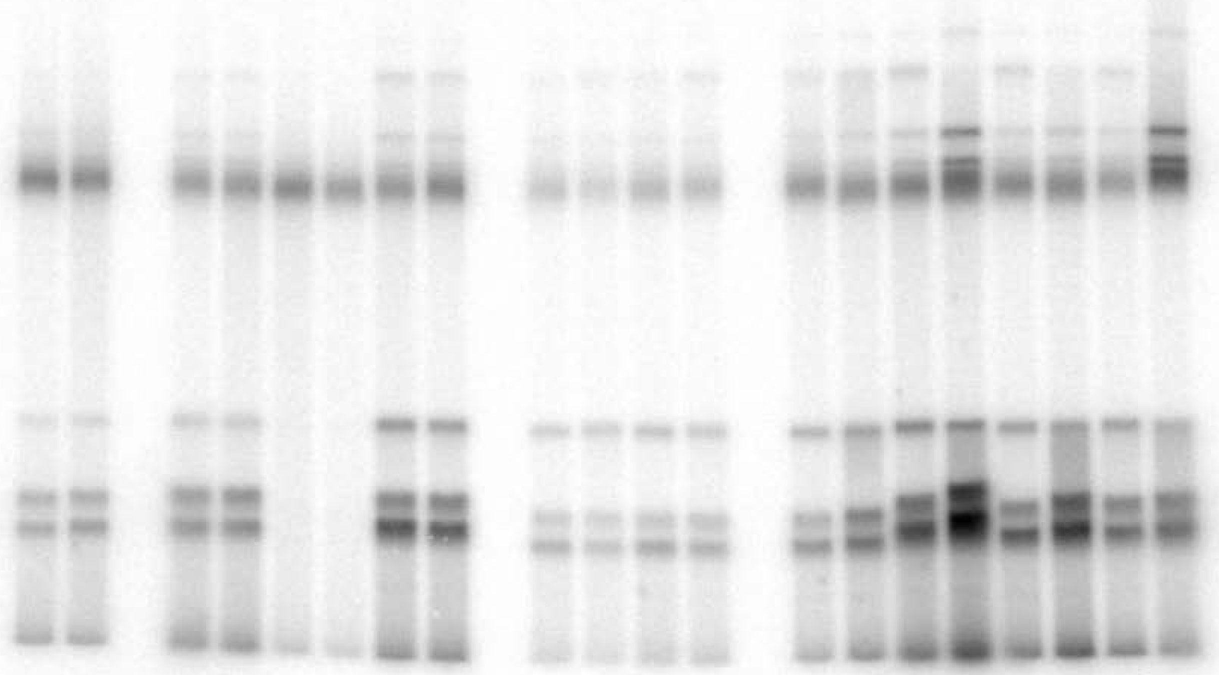

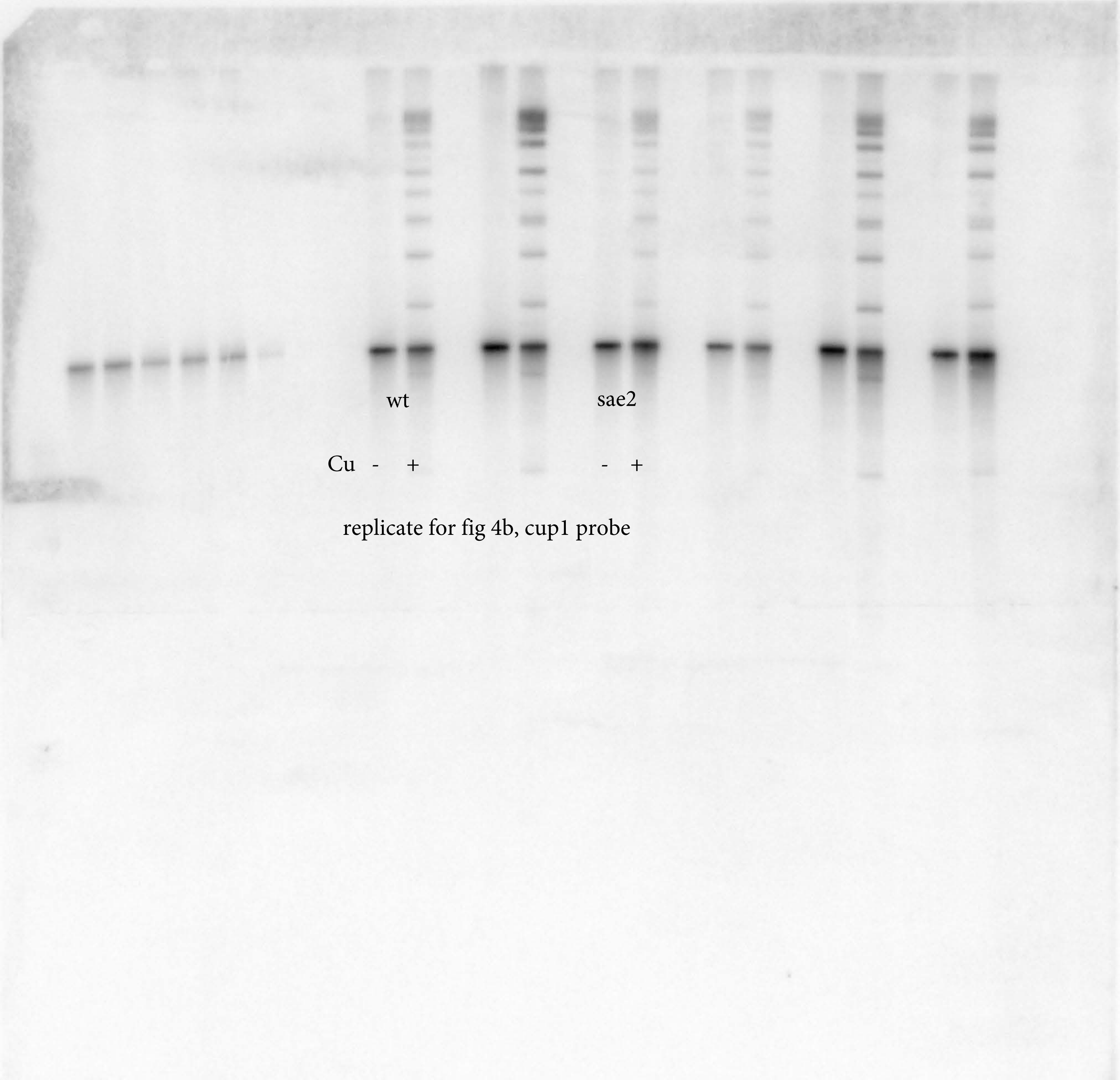
wt
sae2
Cu - + - +
replicate for fig 4b, cup1 probe

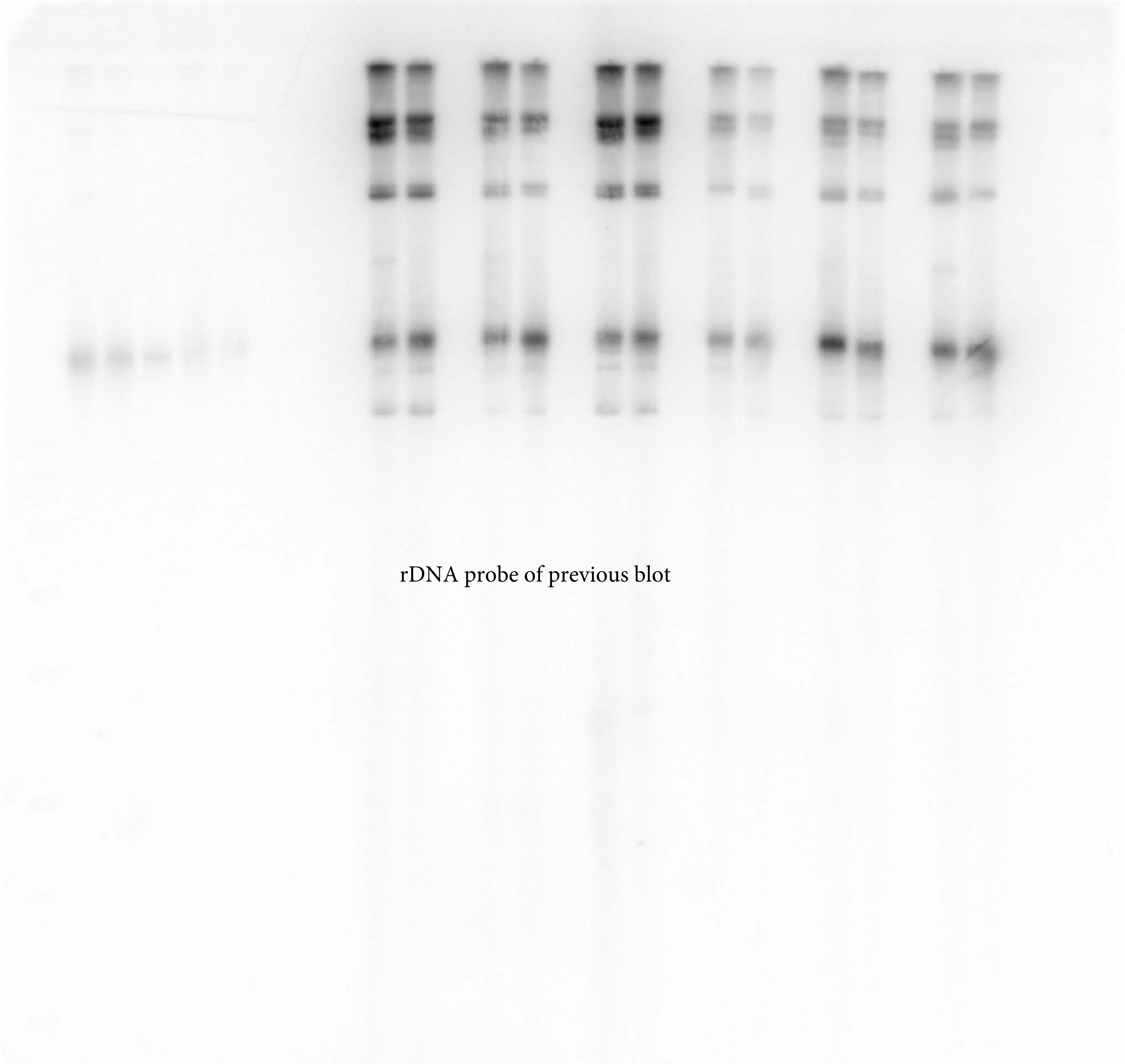

rDNA probe of previous blot

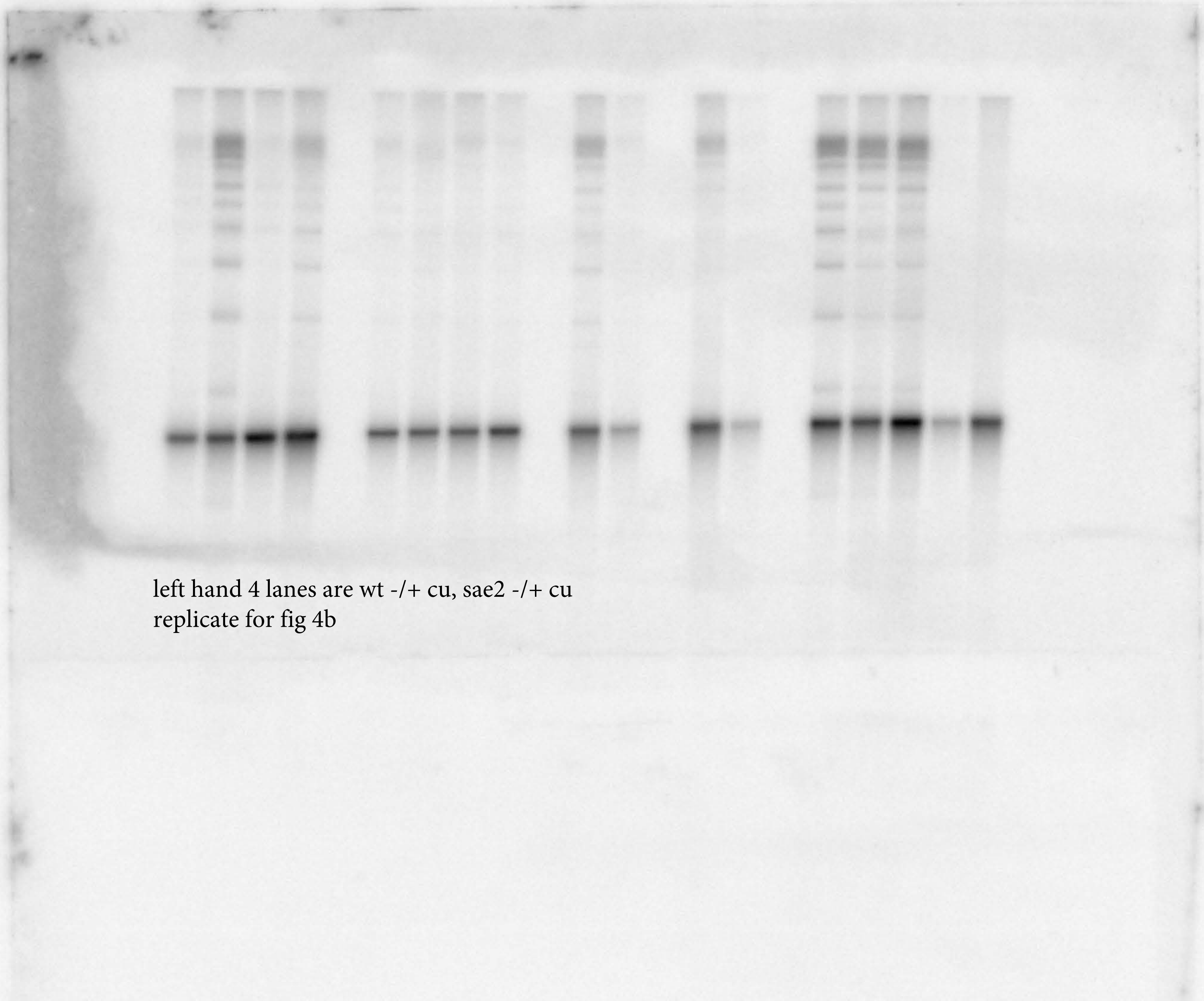

left hand 4 lanes are wt -/+ cu, sae2 -/+ cu
replicate for fig 4b

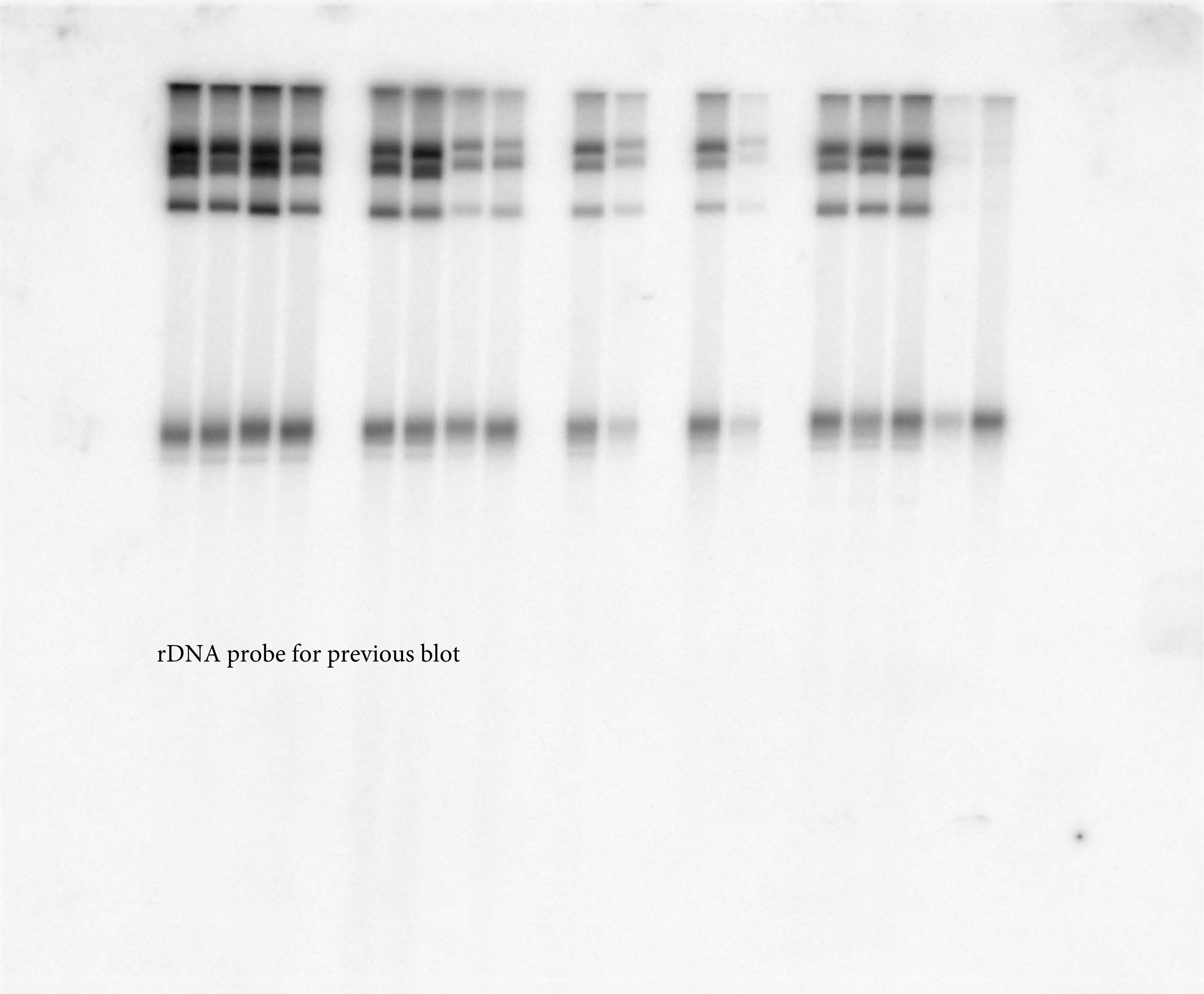

rDNA probe for previous blot

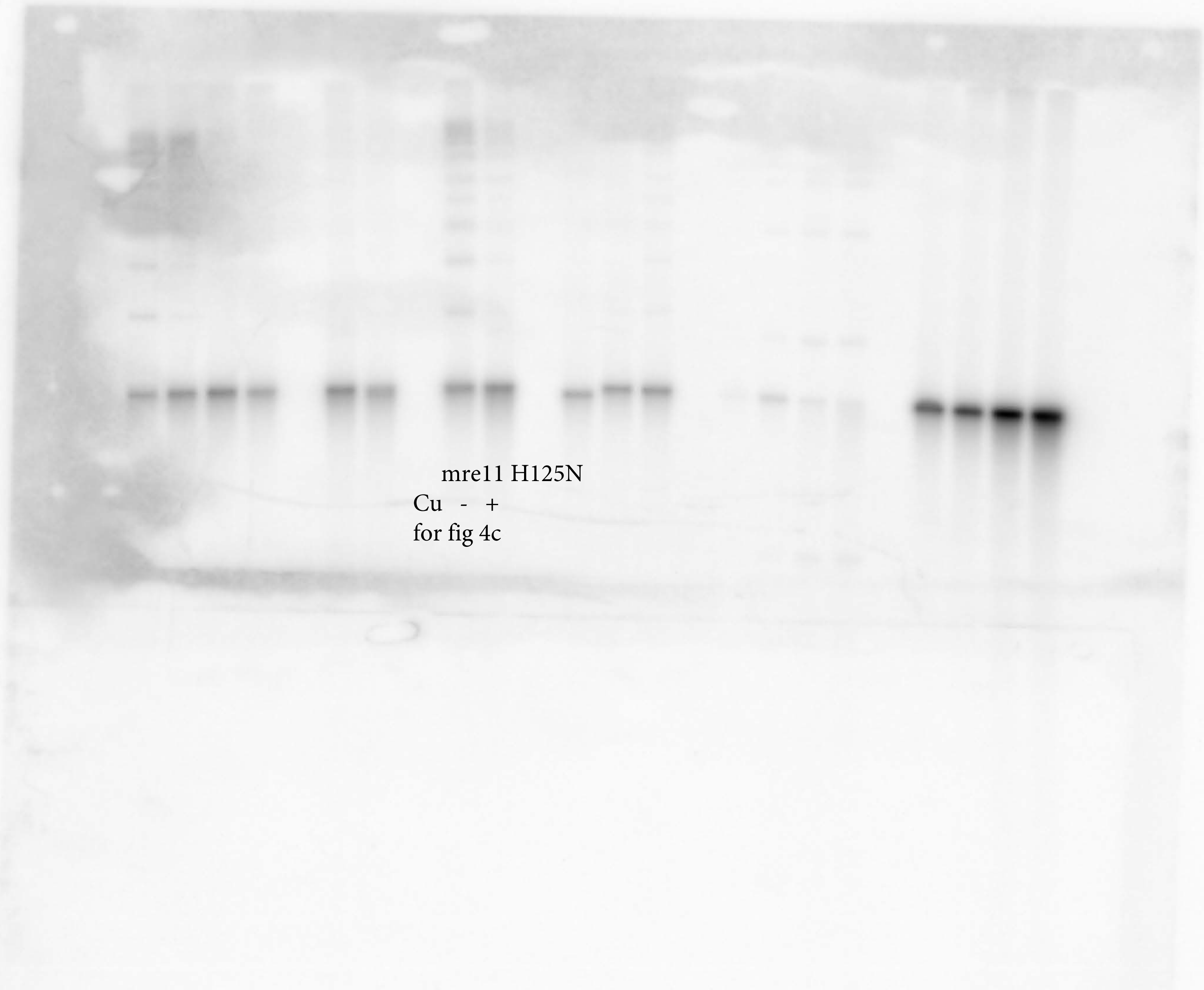
mre11 H125N
Cu - +
for fig 4c

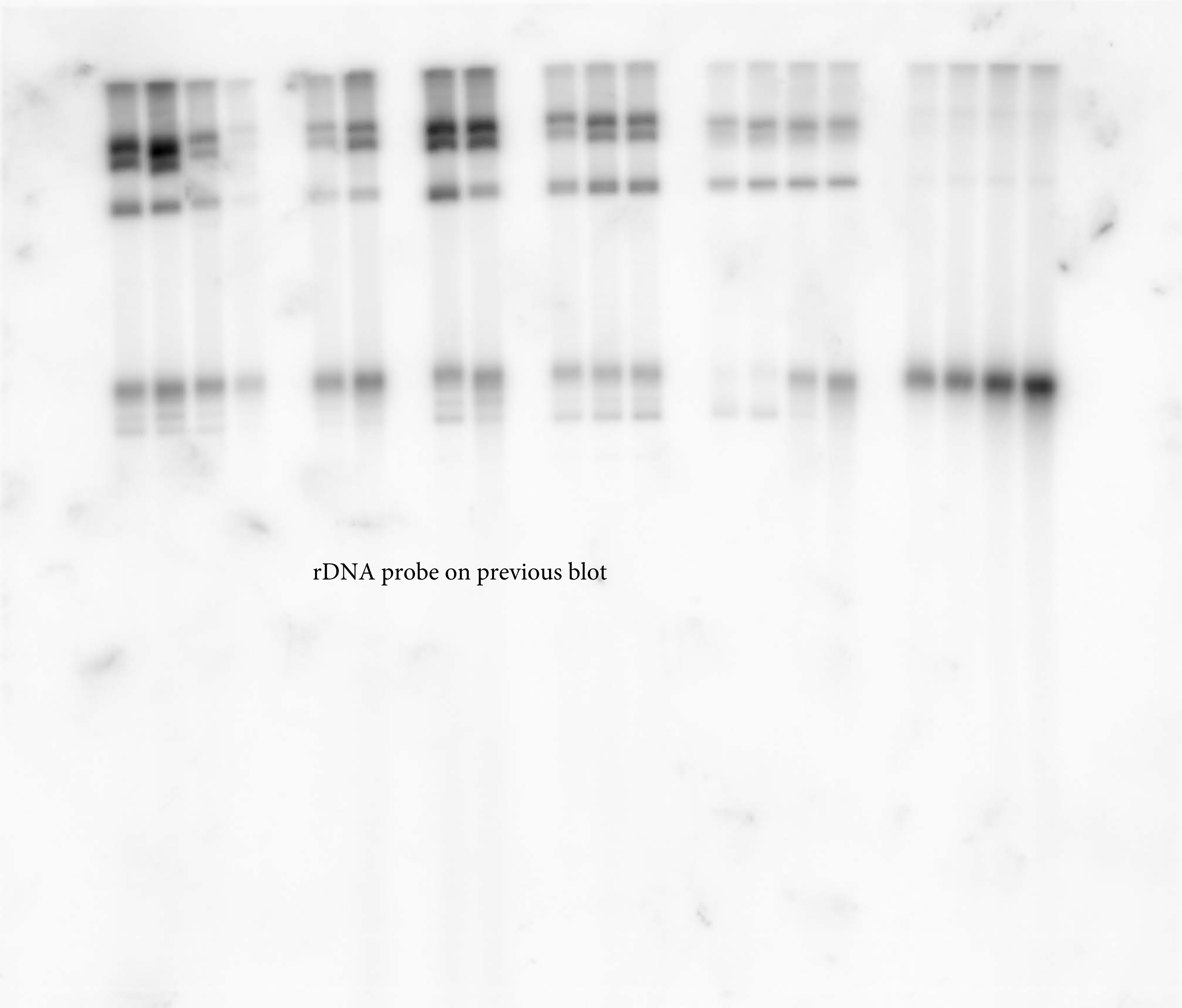

rDNA probe on previous blot

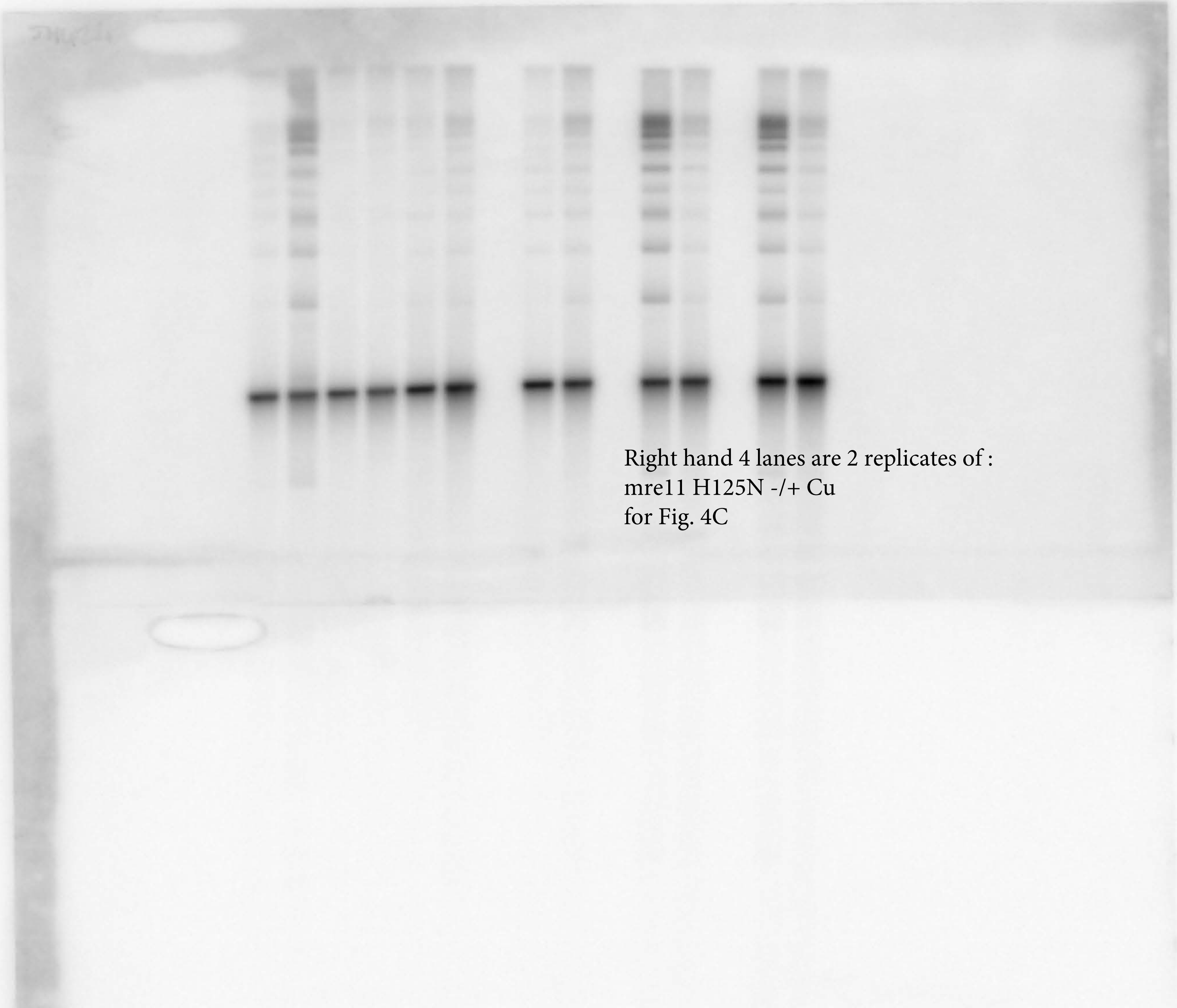

Right hand 4 lanes are 2 replicates of :
mre11 H125N -/+ Cu
for Fig. 4C

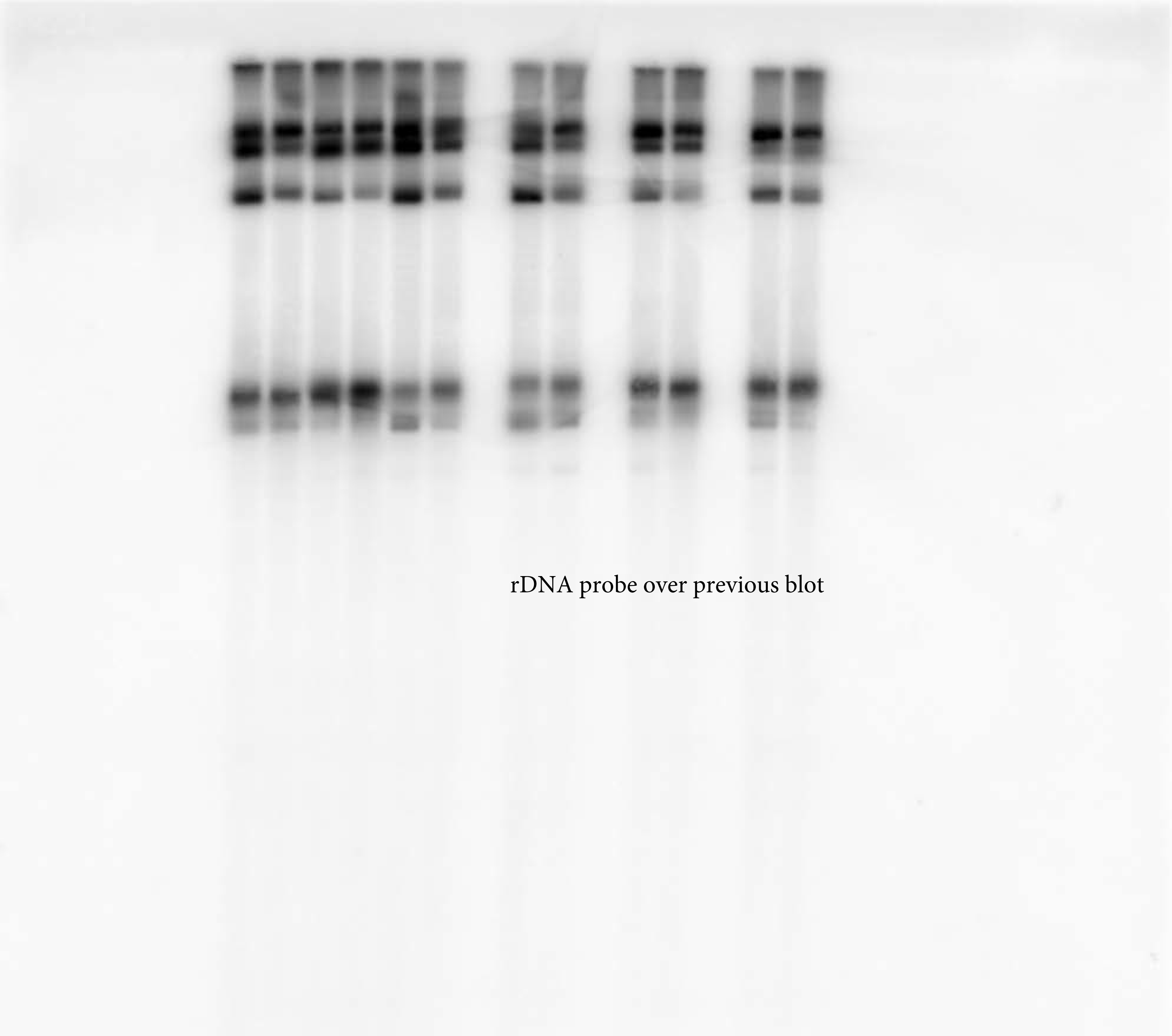

rDNA probe over previous blot

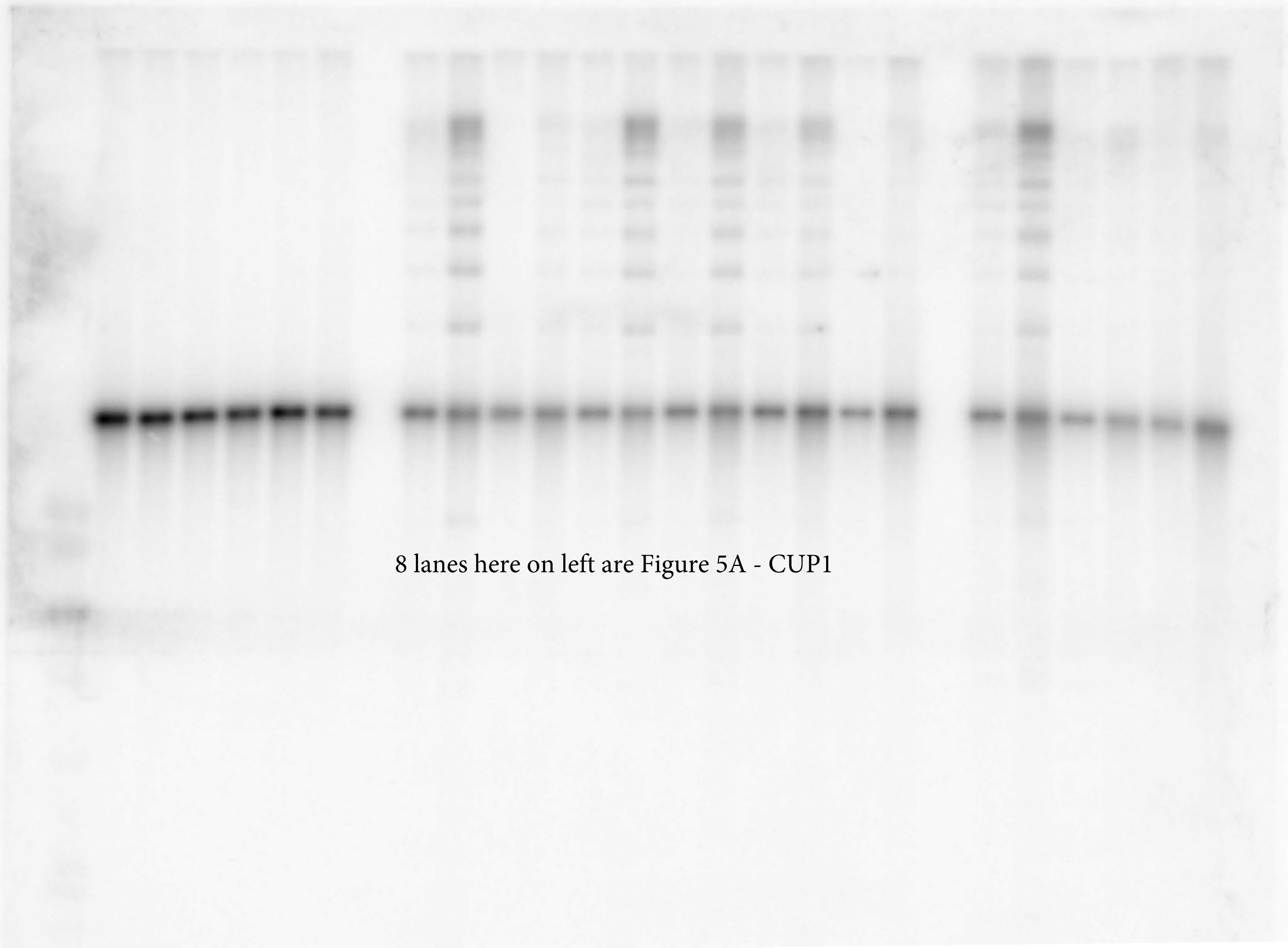

8 lanes here on left are Figure 5A - CUP1

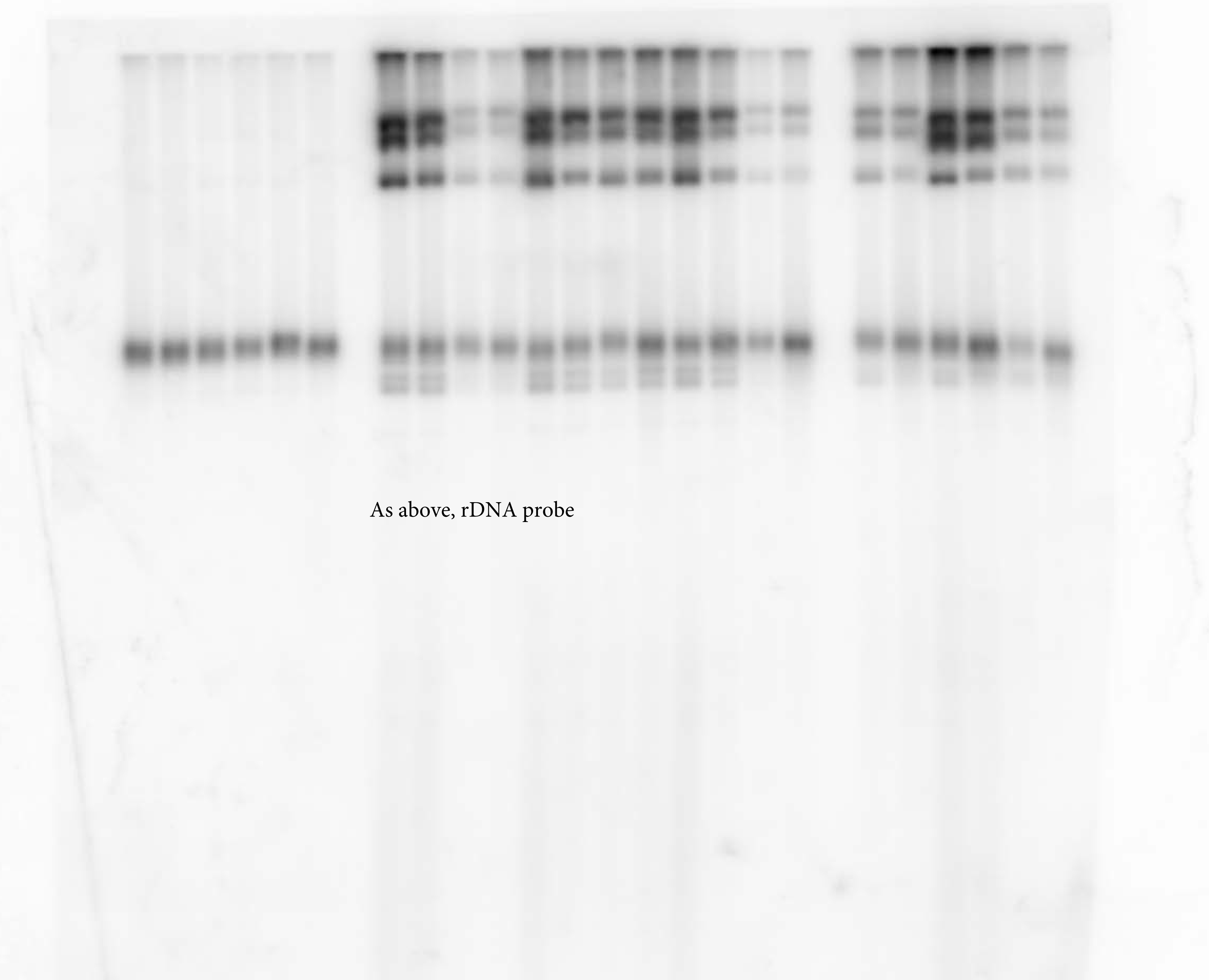

As above, rDNA probe

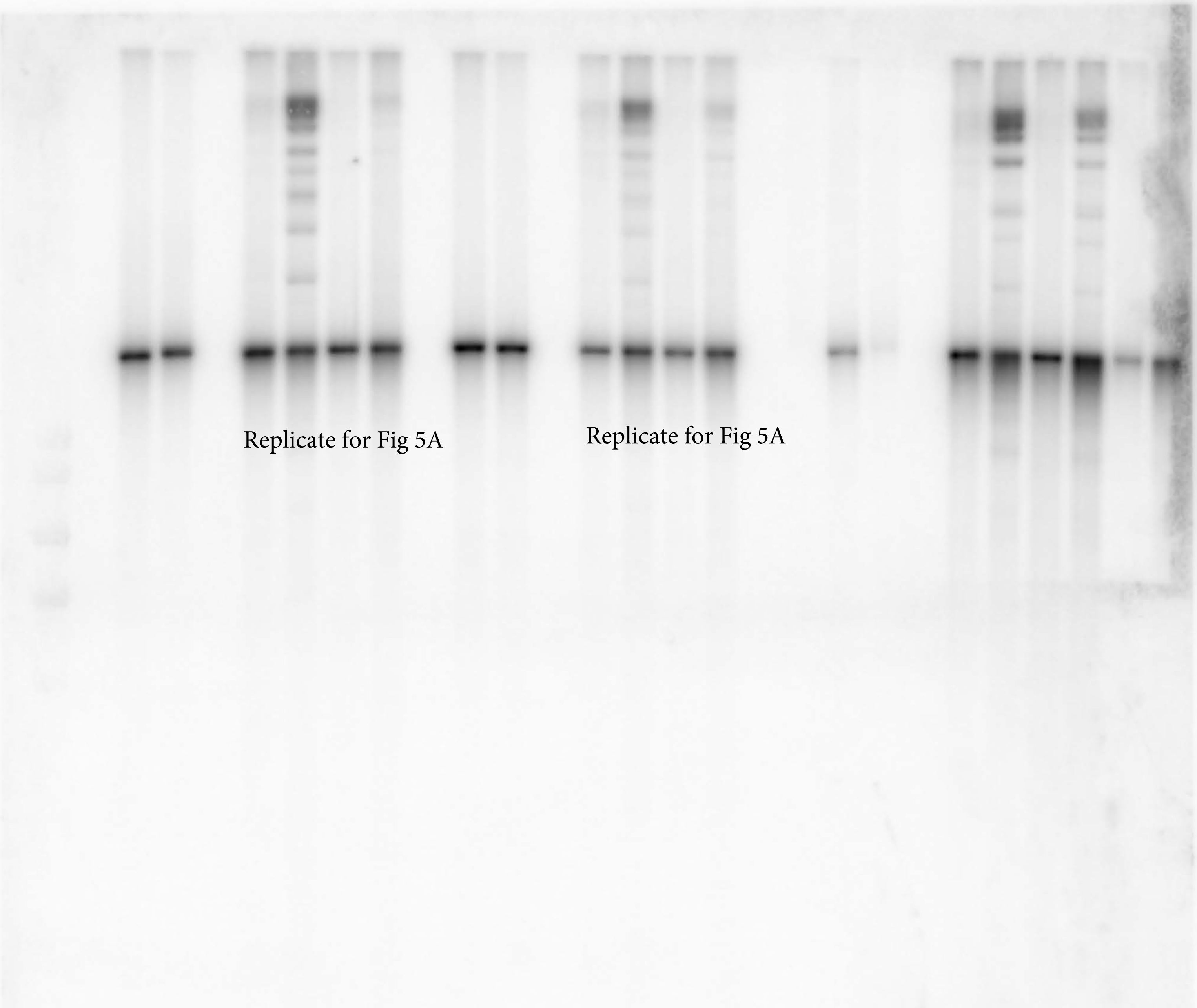
Replicate for Fig 5A
Replicate for Fig 5A

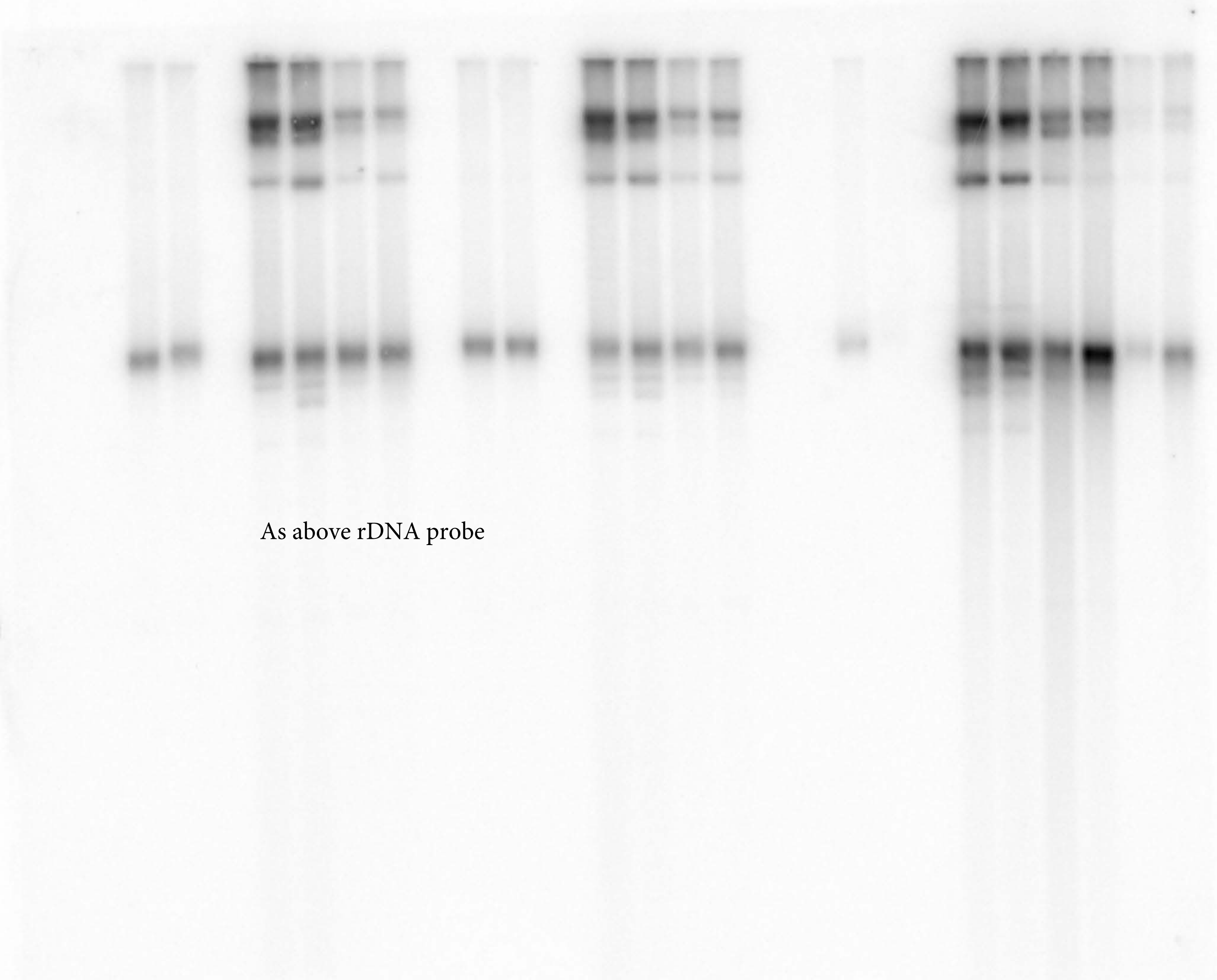

As above rDNA probe

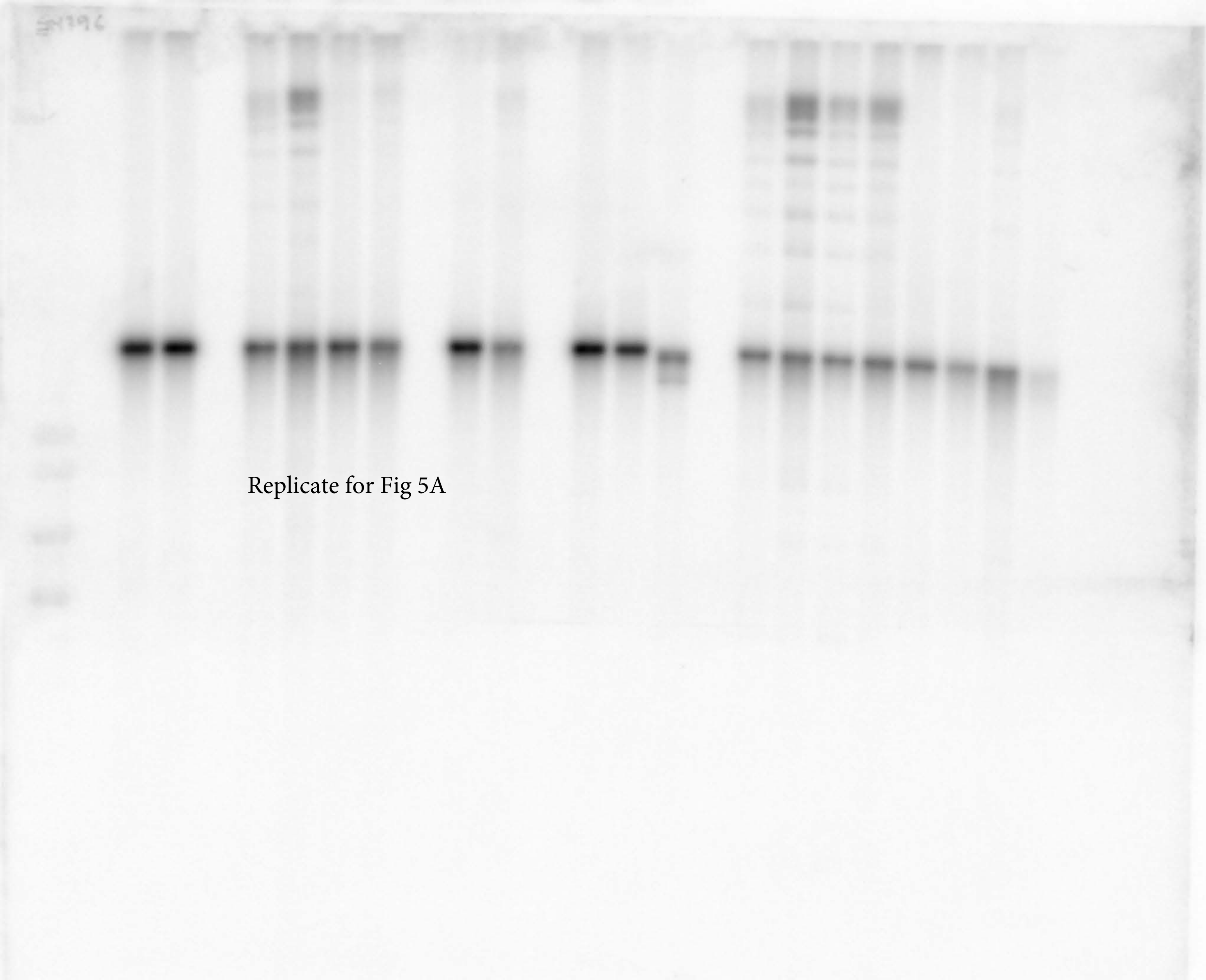

Replicate for Fig 5A

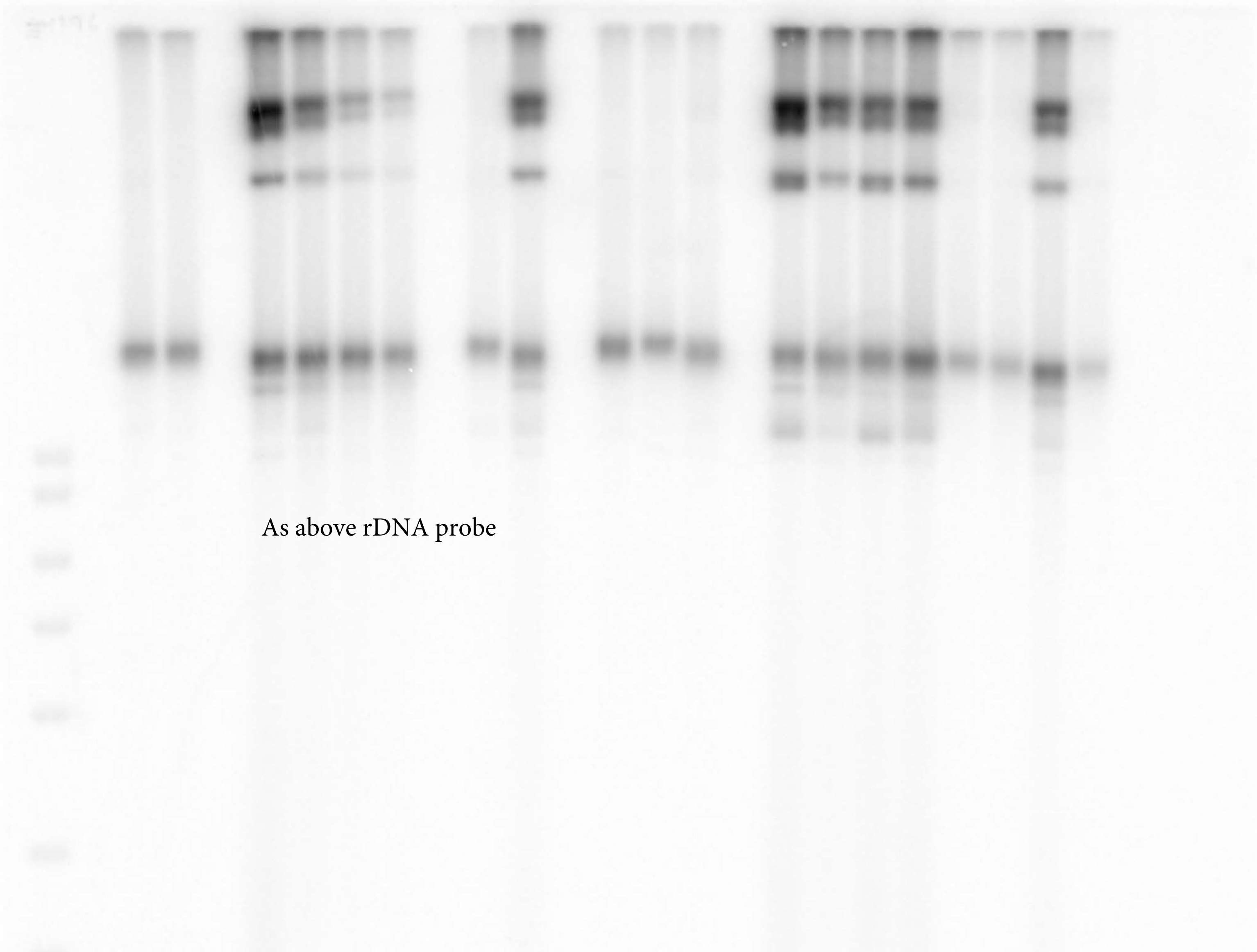

As above rDNA probe

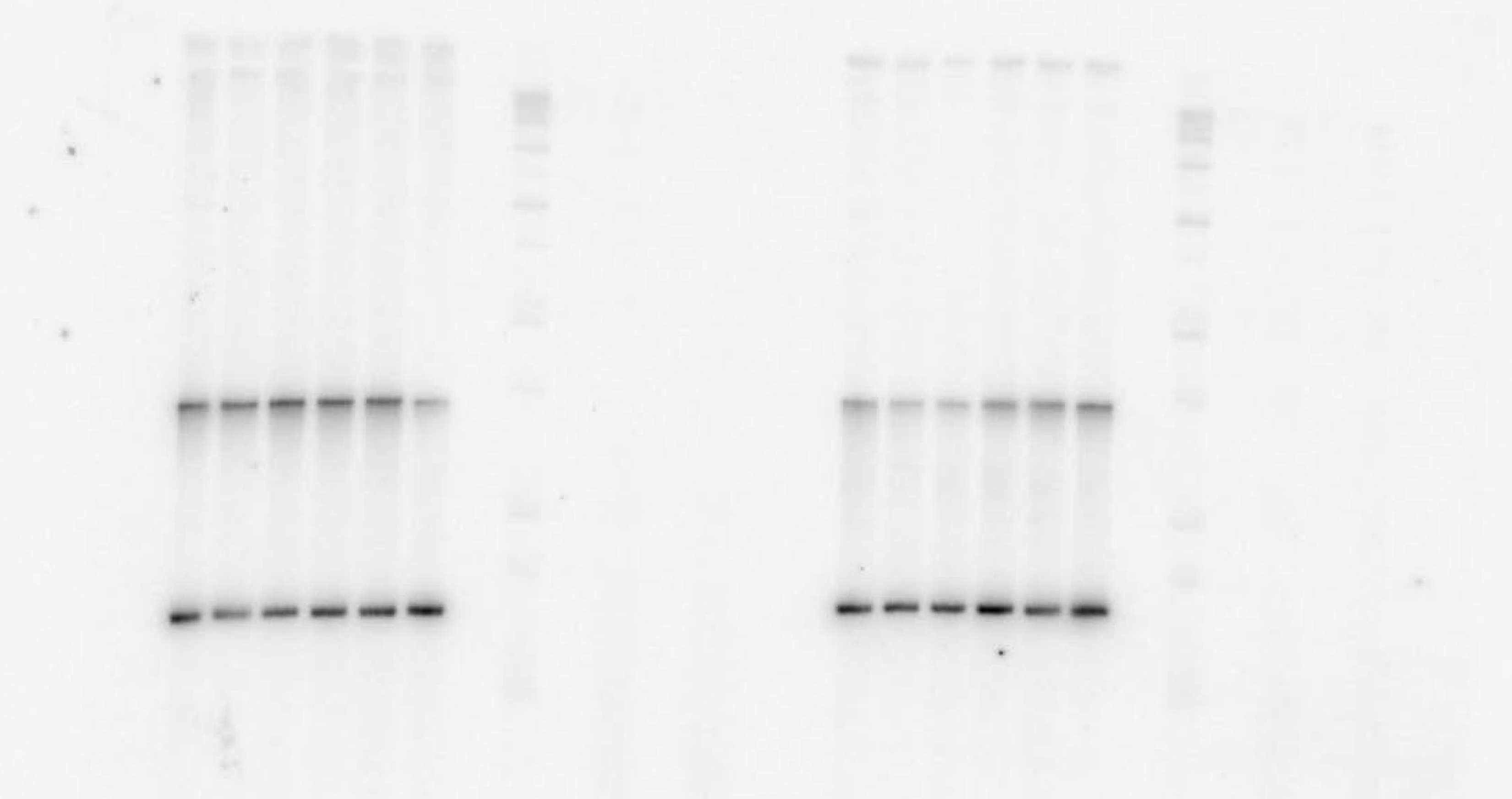

2 replicates for Fig 5D - CUP1 probe

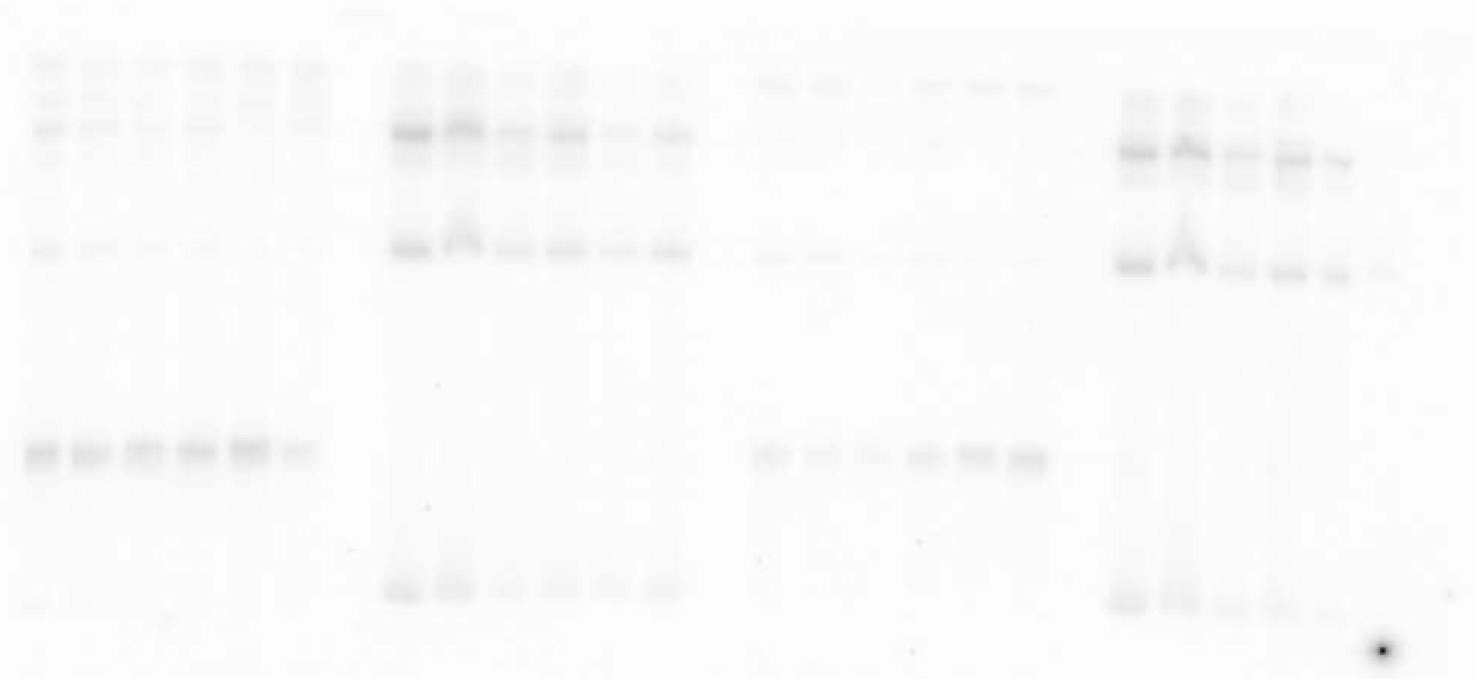

As above rDNA probe

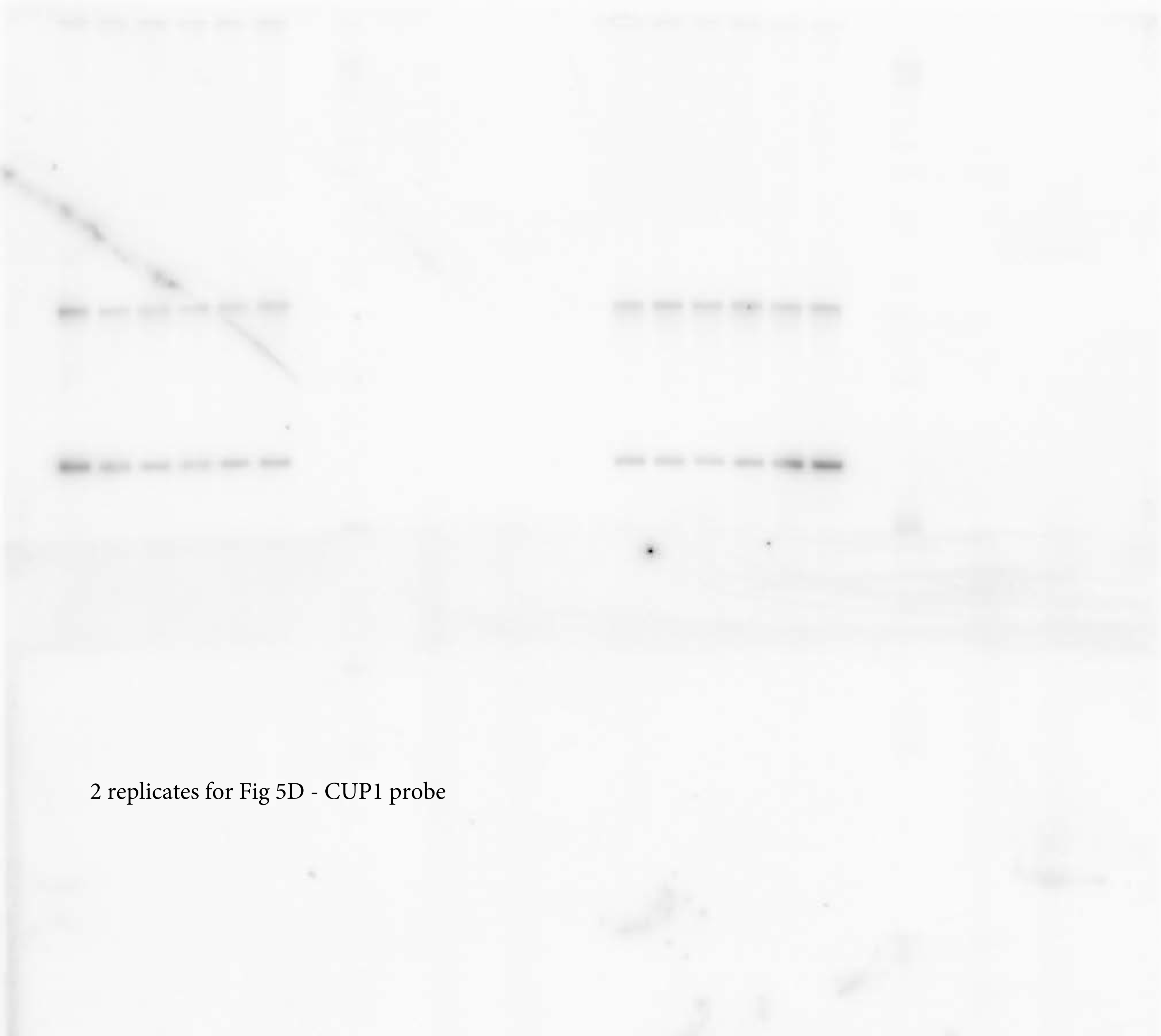

2 replicates for Fig 5D - CUP1 probe

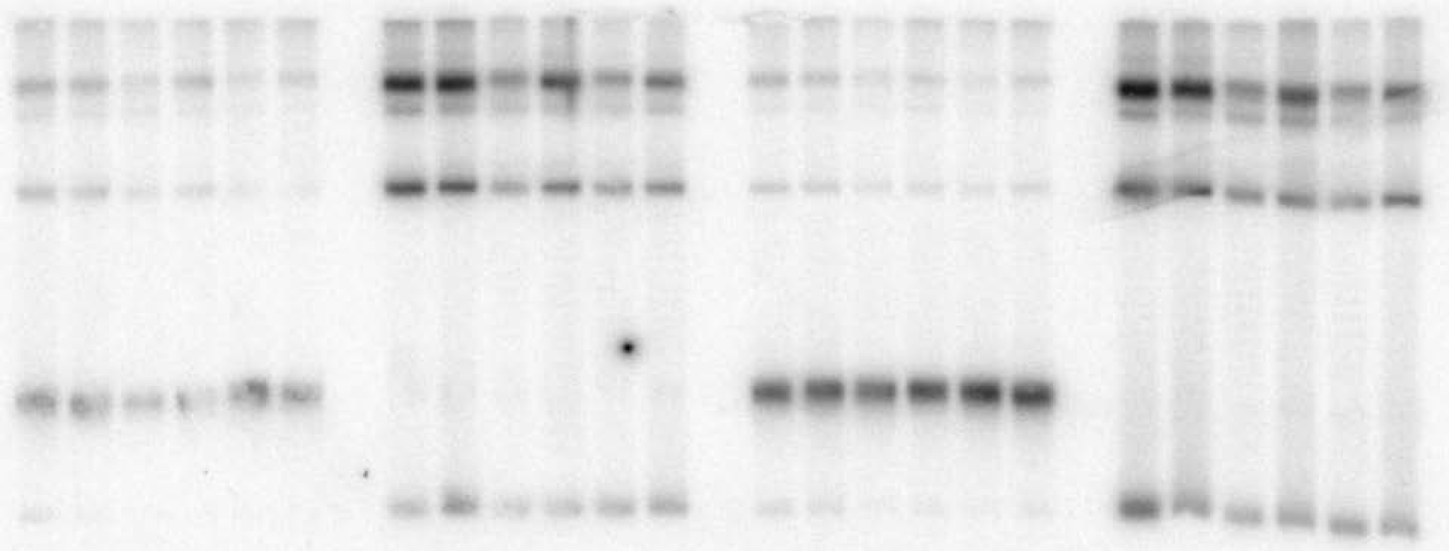

As above rDNA probe

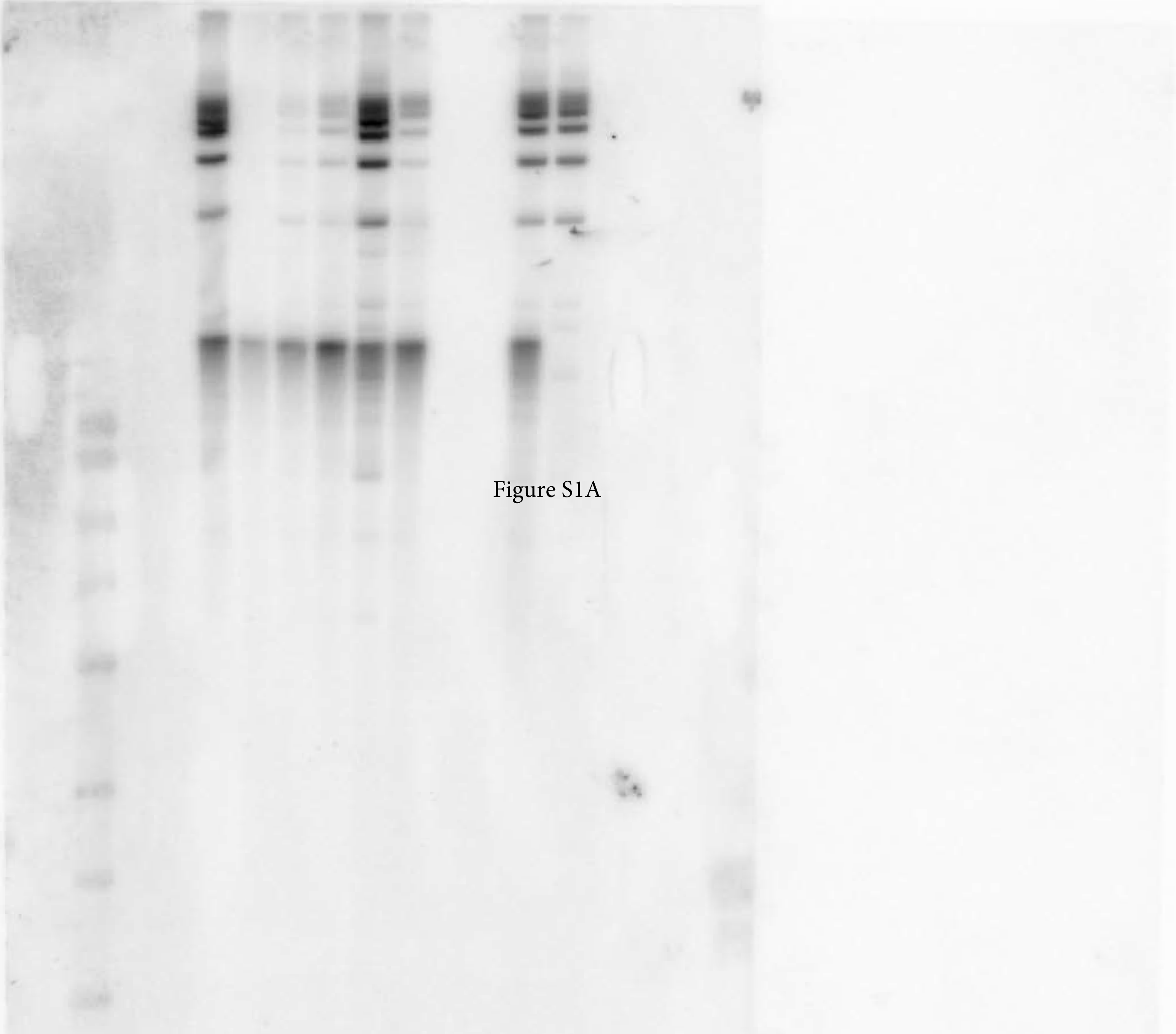

Figure S1A

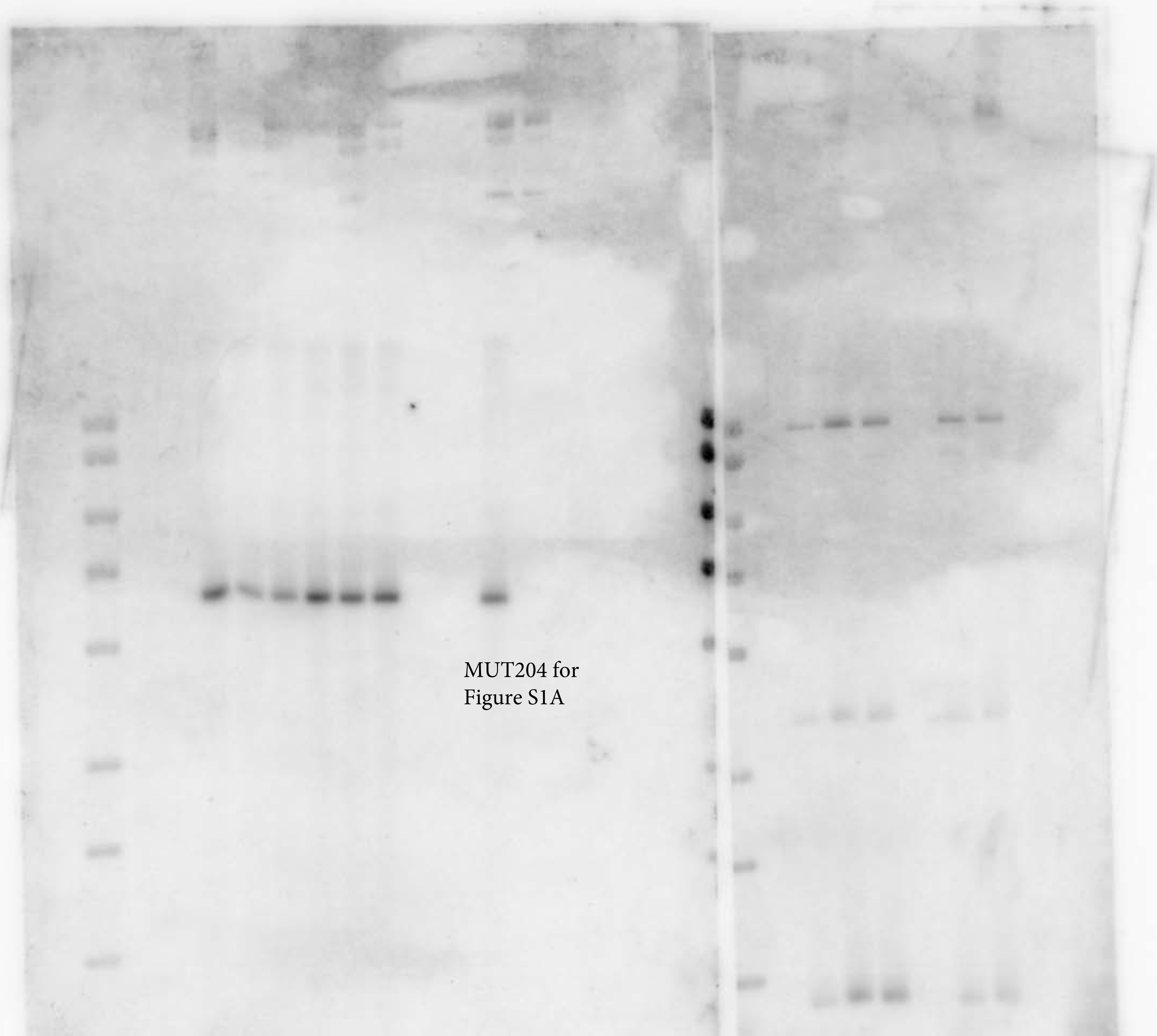
MUT204 for
Figure S1A

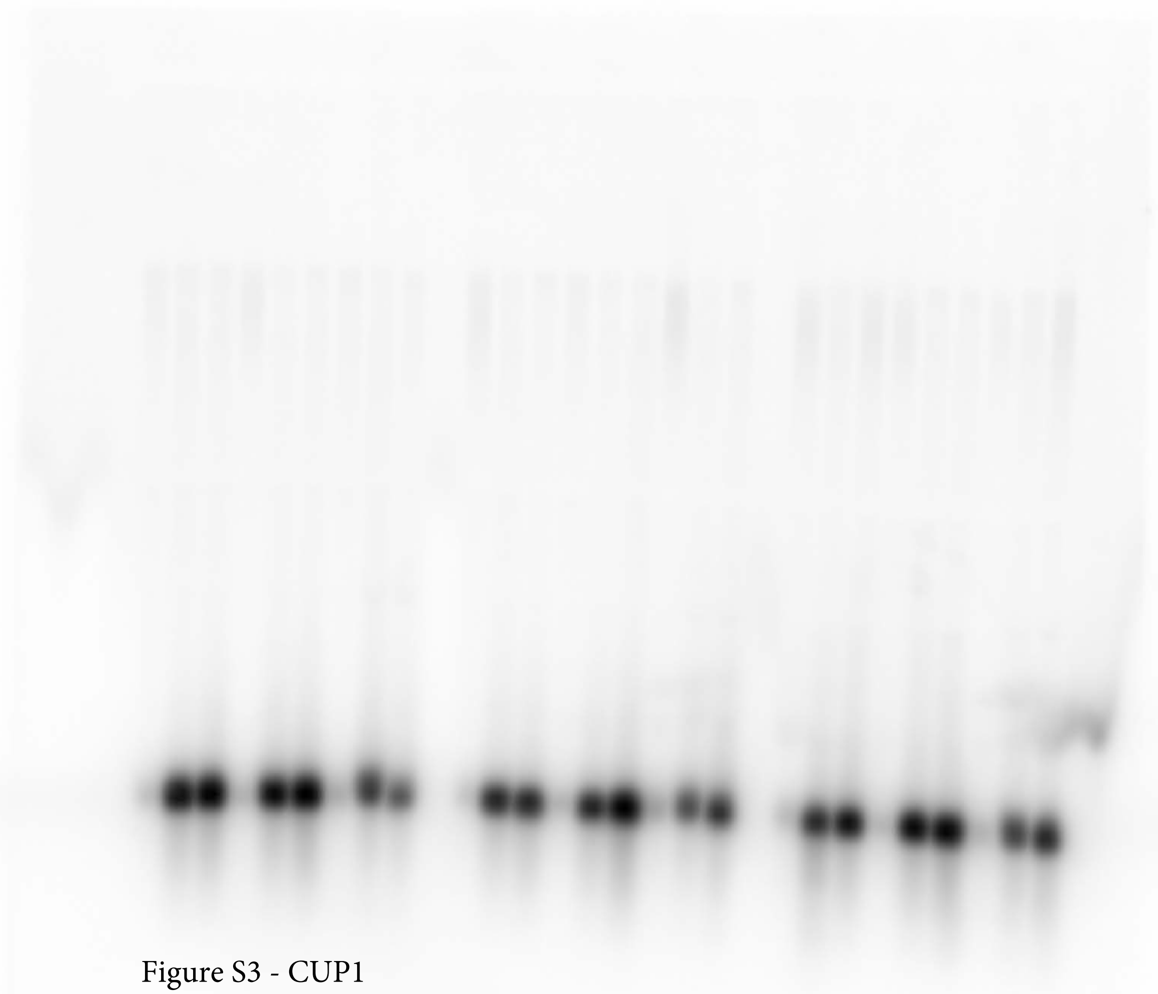

Figure S3 - CUP1
3 replicates, but the last 3 samples in each replicate are for a mutant that is not used in the final manuscript.

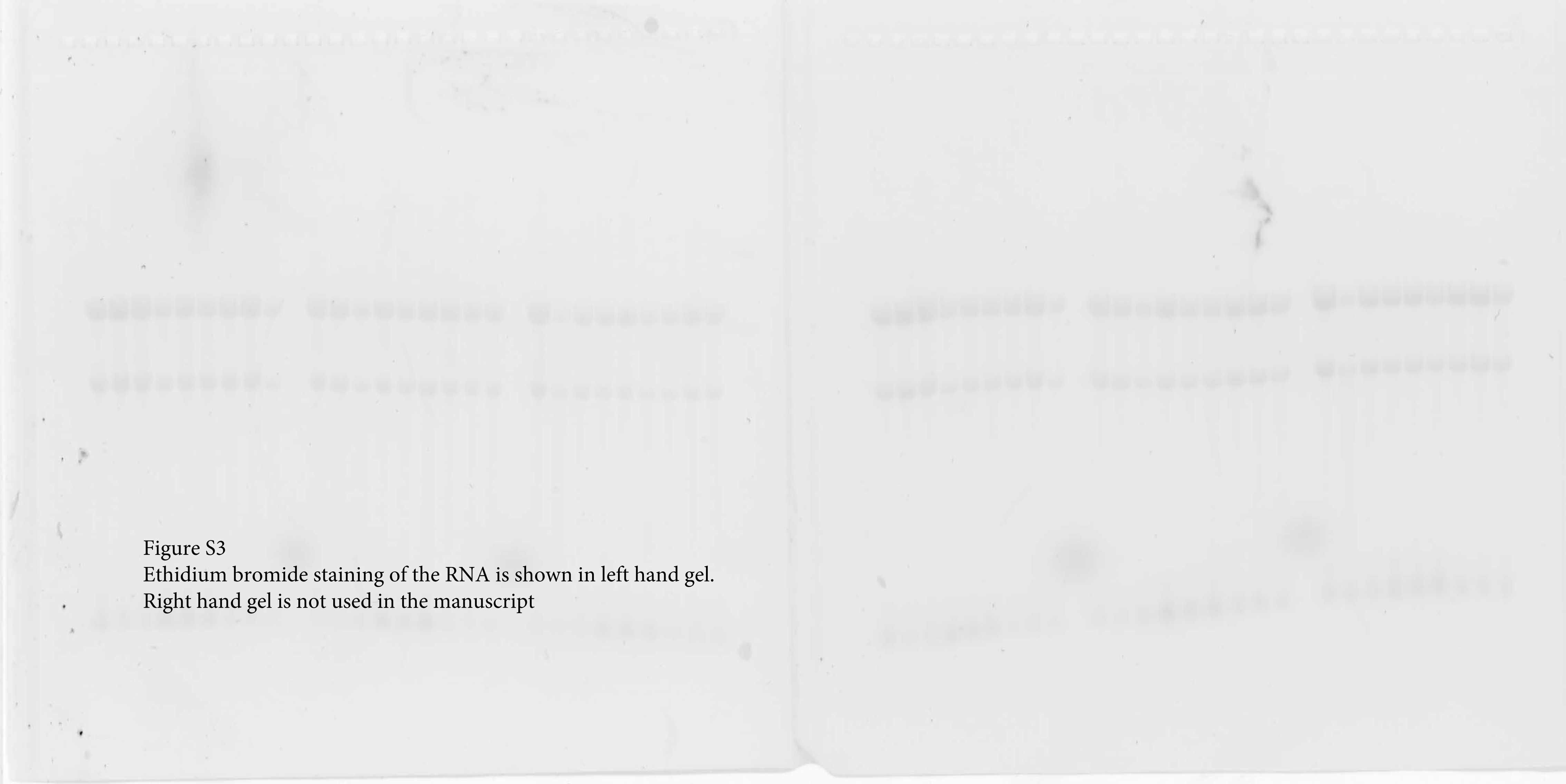

Figure S3
Ethidium bromide staining of the RNA is shown in left hand gel.
Right hand gel is not used in the manuscript

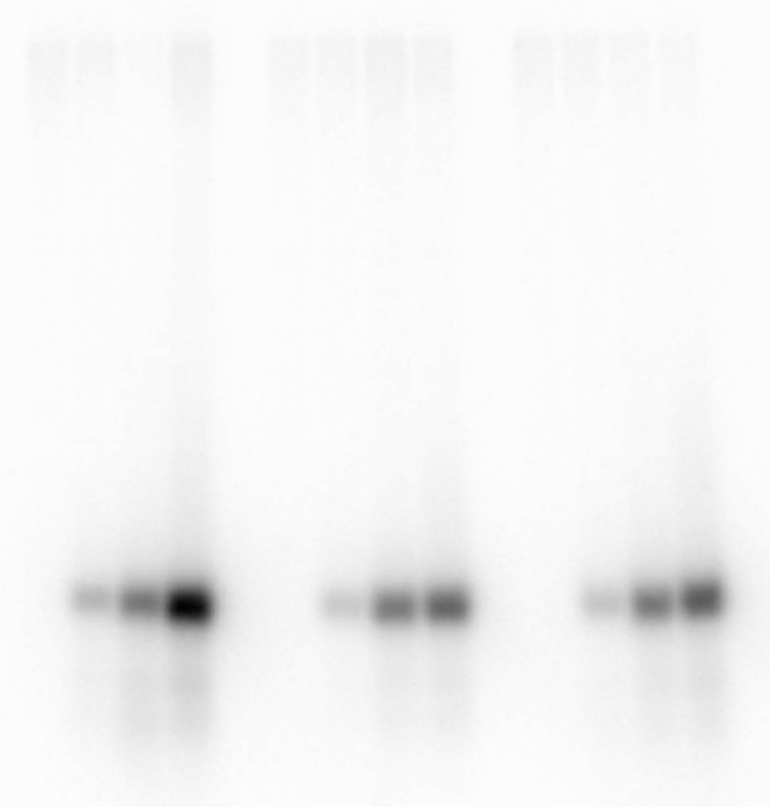

Figure S5 3HA

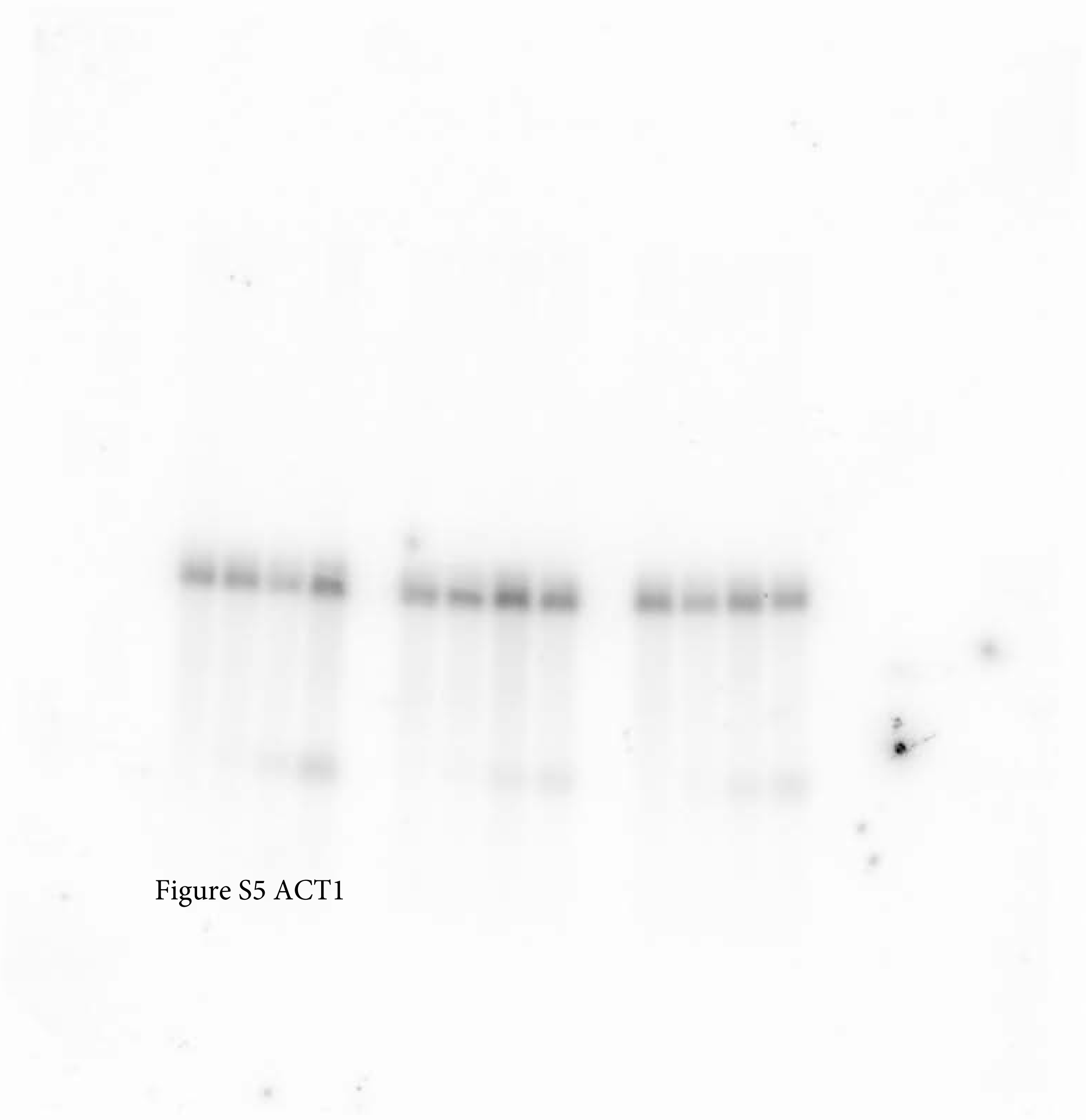

Figure S5 ACT1
