# Supplementary material for: Transcription-induced formation of extrachromosomal DNA during yeast ageing
Source: PLoS Biol. 2019 Dec 3;17(12):e3000471. doi: 10.1371/journal.pbio.3000471 (PMC6890164; doi:10.1371/journal.pbio.3000471)
Supplement: Attachment — Submitted filename: circles paper Response to reviewers 2_RH.docx [file pbio.3000471.s012.docx]

Response to reviewers’ comments

We thank the reviewers for their helpful comments, which have definitely improved the manuscript. We have addressed these comments individually below.

Reviewer #1:

The manuscript by Hull et al. describes the mechanism by which amplification of the CUP1 gene in yeast occurs through formation of extrachromosomal circles (eccs). The same group had shown in 2017 that growing yeast in the presence of copper leads to CUP1 amplification. Now, using newly developed techniques that allow accurate sequencing and quantification, they show that the amplified units are DNA circles formed by homologous recombination repair of double-strand breaks, apparently induced by transcription. The mechanism is very specific to the region being transcribed. Ecc concentration in aged cells depends upon control of segregation, but apparently their formation does not. Ecc formation in ribosomal DNA occurs by a different mechanism dependent on barriers to replication. It is apparent that a low level of CUP1 ecc is also formed by a different mechanism. Thus, there is clearly more than one mechanism of ecc formation. The double-strand break dependence is based on genetic requirements, Sae2, Mre11 and Mus81. The transcription dependence is based on publications by Fogel, who worked on CUP1 expression and amplification in the 1980s and showed that copper induced transcription of CUP1. It is, however, also supported by experiments that used a GAL promoter that showed circles when grown on galactose and not when grown on glucose. These are the same experiments that demonstrate that selection for copper resistance is not required for the amplification.

The authors point out that this seems to be a very specific and robust adaptive mechanism, in that amplification occurs only in those genes to be transcribed in a given environment thus allowing increased expression of needed genes. The relationship of mutation to transcription in other systems has been discussed in this context before.

Indeed. We have tried to focus here on the mechanisms by which amplification can be focused to specific genes rather than the adaptive outcome. In this regard, we had not sufficiently discussed previous work on the relationship between mutation and transcription in other systems, particularly where mediated by R-loops, and have expanded this section in the Discussion (also requested by Reviewer 3).

The manuscript does not make it plain that this mechanism only operates because there is already tandem chromosomal amplification of the CUP1 locus. Thus the generality of the mechanism appears to be limited, and this should perhaps be mentioned.

We have now specifically noted this in the abstract, and also raised the importance of sequence homology in the last paragraph of the Discussion.

The work does not reveal how transcription generates double-strand breaks. That will require a different approach.

Yes – this work is ongoing in our group.

Overall this is a delightfully careful and insightful paper that provides substantial mechanistic insights into a specialized mechanism. The figures, including extended data, are necessary for the conclusions being made.

Reviewer #2:

This manuscript investigates extrachromosomal DNA circle formation at the repetitive CUP1 locus in budding yeast. The formation of eccDNA and its unequal segregation during cell division provides a potential adaptive mechanism for gene amplification in response to environmental stress and is commonly seen in cancer. The authors show that eccDNA formation at the CUP1 locus is pronounced in replicatively aged cells, similar to the well-known age-associated accumulation of rDNA circles in yeast. In contrast to rDNA circles, however, CUP1 circles are only observed at high levels if yeast were exposed to environmental copper, and the authors show nicely that this effect is linked to the transcriptional activation of the CUP1 locus. In addition a sequencing strategy is presented that allows recovery and comparison of different eccDNA species. The formation of CUP1 circles is shown to depend on the resection factors Sae2 and Mre11 and the recombinase Mus81, indicating a role of the homologous recombination in circle formation.

The manuscript is clear and well written and the presented experiments are for the most part conclusive. My main concern is the attempted normalization between sequencing results using endogenous 2-micron plasmid ratios as the denominator. Overall, this manuscript presents an intriguing new system that should allow the mechanistic dissection of stress-induced eccDNA formation and is expected to be of substantial interest to the field.

Major points:

1. The normalization approach using 2-micron requires more detailed description in the methods. It is difficult to understand how the 2-micron plasmid ratio was used to normalize the data. On a more general note, I am also not convinced that 2-micron plasmid levels, which are themselves changing (e.g. btw wt and spt3), are a reliable signpost for normalization. How can the authors be certain that apparent changes in the abundance of other eccDNA species are not secondary to changes in 2-micron levels under different experimental conditions? Fluctuations in 2micron levels could in principle explain some of the unexpected differences between samples. I think a simple solution to this problem would be to spike a bacterial plasmid into the sequencing samples and use those easily identifiable sequencing reads as a normalization factor.

We apologise – this method was not well enough explained so we have added a section at the end of the Materials and Methods to address the normalisation. Importantly, because we have both DNA-seq and REC-seq for each sample, we can control for variations in 2µ level between samples, and in reality our strategy normalises eccDNA to chromosomal DNA, it just uses 2µ as an intermediate that is detected in both data types. The normalisation is depicted in Figure S4; in this example (wt vs *spt3*Δ), the total DNA seq reveals the abundance of 2µ to be ~2-fold lower in *spt3*Δ (Fig. S4A). After normalisation is applied to the eccDNA in the REC-seq libraries, the 2µ level is equivalently lower in *spt3*Δ than wt as in the total DNA (Fig. S4B).

Nonetheless, we agree that spiking in a bacterial plasmid would be a better approach and plan to use this in the future, primarily because the 2µ reads are so dominant in the libraries.

2. The sequencing analysis shows sharp boundaries for the CUP1 derived eccDNA but I did not expect the boundaries to lie in the neighboring RSC30 gene. Are there obvious sequence homologies that could explain these boundaries?

Yes – the *CUP1* repeat sequence overlaps the *RSC30* gene. The boundaries of the circularised sequence fit precisely with the boundaries of the repeated region as far as we can tell. We have noted this in the text.

3. The authors imply that CUP1 circle formation is the result of DSB-stimulated loop-out of CUP1 copies by homologous recombination. They also draw parallels with rDNA circle production but those circles can form without loss of the chromosomal repeats (e.g. Mansisidor et al, Mol Cell 2018). The presented loop-out model would predict that the chromosomal CUP1 locus should show signs of copy number loss, which should be easily detectable by marker loss or Southern given the relatively small size of the CUP1 locus, in particular when analyzing circle formation in the Gal-inducible system.

This is an interesting point, and we see potential for both conservative and non-conservative repair of the DSB after eccDNA excision. We have added a paragraph to the Discussion (at the end of the *A mechanism for the formation of recurrent eccDNA* section), and a further panel to Figure 6 exploring this question.

Minor points:

1. I assume the CUP1 plasmid in Figure 5D contains an XhoI site? This could be indicated in the figure legend to avoid confusion why a circular DNA element is sensitive to ExoV.

Yes - we have now noted this in the figure legend.

2. The last section of the discussion feels overly speculative. The potential effect of TATA boxes is certainly worth exploring but the relevance to the current work appears tangential. The presented mechanism implies a need for flanking homologous DNA sequences, which are not expected to be a common enough genomic feature around TATA-driven genes to warrant the broad statements of this paragraph.

We agree that this was rather speculative. We have removed the explicit statements regarding TATA boxes and noted simply that chromatin state can differentiate house-keeping and inducible genes, which was our major point. In the yeast genome, such flanking sequence homologies are indeed rare, however as reviewer #3 notes this could be provided by repetitive elements in higher eukaryotes, and we have altered this paragraph to discuss the importance of local sequence homology. We appreciate that the high copy *CUP1* sequence is unusual, which was why we performed the analysis of the 3 copy *CUP1* system (Figure 2F,G); such low copy repeats are much more common in eukaryotic genomes.

3. Please make all code available on GitHub.

We have deposited the code for de-duplicating the REC-seq reads and added a citation in the Materials and Methods. All the other bioinformatics analysis was performed directly within SeqMonk. We have also added the description for 2µ read normalisation as noted above.

Reviewer #3:

The current study uses the CUP1 tandem-repeat locus as a model to examine environmentally stimulated accumulation of extrachromosomal circular DNA (eccDNA) in aging yeast cells, which has potential implications for analogous eecDNA formation in higher eukaryotes. The authors used the mother-enrichment program to obtain a population of aged cells and demonstrated an association between the levels of CUP1 eccDNA accumulation and transcription. Transcription from the endogenous promoter was regulated by copper addition or by galactose addition when a mutant repeat unit was fused to the GAL1 promoter; the effect of transcription was locus specific. As with ERCs (ribosomal DNA circles), the accumulation of eccDNA in aged cells required their asymmetric segregation to mother cells. Importantly, the authors used specific genetic backgrounds to examine the mechanism of eccDNA accumulation. Accumulation in the absence of DNL4 excluded NHEJ and the lack of accumulation in the absence of the initiation of DSB end processing (sae2 or mre11-nd mutants) implicated homologous recombination (HR). A requirement for Mus81 (but not Yen1 of Slx4) is consistent with an HR-based mechanism that requires the processing of nick-containing branched intermediates. Interestingly, eccDNA accumulation has variable genetic requiremnents relative to ERC accumulation, which is related to the strong replication block in rDNA. The manuscript is well-written, the data are robust, and the results are important and of broad general interest. Below are comments that should be addressed when revising the manuscript.

1. If the frequency of DSB formation is high enough to cause a growth defect in a rad52 background (which is not a direct demonstration of DSBs), then it should be possible to physically “see” the broken chromosome on a CHEF gel. If the authors haven’t tried this, they should. eccDNAs may be only a very rare outcome of DSB repair, but this doesn’t make them any less interesting.

We agree that direct detection of the DSB would be helpful, and have tried this experiment. Unfortunately, we could not detect any galactose-specific bands in *P_GAL1_-3HA rad52*Δ, or in fact any bands of the predicted size for Chromosome VIII cleaved at the *CUP1* locus.

We take the reviewer’s point that the growth defect in the absence of Rad52 does not actually prove that a DSB is present; this indicates DNA damage, which may be an irreversibly collapsed replication fork, or a strand break or blockage. It is the requirement for Sae2 and Mre11 that suggests to us that some proportion of this damage is in the form of DSBs that can then be processed to eccDNA. Therefore in describing the cell growth experiment, we now refer to DNA damage rather than DSBs.

2. The authors need to provide more info about the pGAL1-3HA construct (consulting the reference cited was not much help). When the CUP1 coding sequence is deleted, how much of the repeat unit remains? Presumably most of it, but this should be stated.

Yes, it is only the *CUP1* promoter and ORF that is replaced (477bp out of 1990bp of *CUP1* repeat). We have added a schematic drawing of the structure of this construct in Figure S1B.

3. Following DSB induction in yeast, repair is delayed but not prevented in the absence of MRX/Sae2. Does this imply the presence of damage at the ends of spontaneous DSBs? The authors should comment on this.

Yes – this is an important point which we now raise in the first section of the Discussion.

4. There have been MANY studies of transcription-associated recombination (TAR) in yeast, mostly from the Aguilera lab, most of which results from the persistence of R-loops during head-on conflicts between replication and transcription. The Cimprich lab has shown a similar connection in mammalian cells. These studies should be more prominently highlighted as they may well be directly relevant to eccDNA formation.

We have substantially expanded the section on R-loops in the Discussion including work from both of these groups.

5. The production of eccDNAs by the current mechanism requires pre-existing direct repeats, which are very rare in the yeast genome. In higher eukaryotes flanking repetitive elements (e.g. LINEs) may be the drivers.

We agree completely and have noted this in the last paragraph of the Discussion.

6. The papers the authors cited for TopIIbeta-mediated DSB formation, which is specifically related to transcription initiation in mammalian cells, may not be relevant in yeast.

We agree that this was perhaps too speculative and have removed this suggestion. We have re-written this paragraph in response to comments by reviewers 2&3.

7. Please note that Tom Petes’ lab has also shown that transcription stimulates recombination (in the form of CNV) at the CUP1 locus (Genetics 2017).

Absolutely – this should have been included. We have added this citation in the last paragraph of the introduction where the transcriptional stimulation of *CUP1* recombination is introduced.

Reviewer #4:

This ms. explores an important problem concerning the generation of eccDNA in budding yeast. They have modified the methods used to measure eccDNA and have made several interesting observations on the copper gene multiple tandem array. The work is very well presented (for the most part) and will be an important contribution to the literature.

Please address the questions at the end of each section from Pg. 4: The genome-wide profile of eccDNA was more affected by copper treatment in these cells, likely due to greater copper stress, and many eccDNAs accumulated to lower levels (Figure 2G). However, edgeR analysis (Where is the edgeR data?) of differential accumulation across 3 biological replicates revealed that the significant regions most over-represented in the copper-treated sample all derive from the CUP1 locus. Additionally, Ty-element eccDNA was under-accumulated in copper treated cells (was this significant or not?), while sub-telomeric eccDNA was over-accumulated though by a very small amount (again, was this significant? - what is the metric here “a very small amount”?).

We were not clear enough here. On this panel all 100bp bins in the genome are plotted in grey, the ones called as significant (p<0.05) by edgeR are highlighted in blue. We have added a key to the figure panel to make this explicit. We circled and labelled prominent clusters of significantly different bins called by edgeR as belonging to particular classes of element – many Ty element bins were significantly different. We have improved the description of this figure in the manuscript, and noted the average over-accumulation for sub-telomeric sites in the text (0.3 log_2_ units).

In Figure 5A and B: The figure legend is little confusing. Are the two blots exactly the same experimental conditions and the data from each is then represented in the right side of scatter plot of 5B? If so, consolidate and leave out the left side of 5B (the same can be done in other figures if applicable).

The reviewer is correct – we have removed the blot from Fig. 5B as suggested and replaced with quantification of ERCs as in other figures. Figure 3 had a similar problem and has been re-arranged in the same manner.

In Figure 6 and in the discussion, it is not at all clear why propose a gap in the circle at the end of the process? Trimming and ligation, as shown in earlier steps can be invoked without an obligatory gap.

We had left the gap in the circle to indicate where the break originally occurred, but agree that this is confusing as the ligation should be rapid following cleavage of the D-loop. We have therefore removed the gap.
